# Functional brain mapping using whole-head very high-density diffuse optical tomography

**DOI:** 10.1162/IMAG.a.54

**Published:** 2025-06-20

**Authors:** Morgan Fogarty, Sean M. Rafferty, Zachary E. Markow, Anthony C. O’Sullivan, Calamity F. Svoboda, Tessa George, Kelsey King, Dana Wilhelm, Kalyan Tripathy, Emily M. Mugler, Stephanie Naufel, Allen Yin, Jason W. Trobaugh, Adam T. Eggebrecht, Edward J. Richter, Joseph P. Culver

**Affiliations:** Imaging Science Doctoral Program, Washington University in St. Louis, St. Louis, MO, United States; Mallinckrodt Institute of Radiology, Washington University School of Medicine, St. Louis, MO, United States; Western Psychiatric Hospital, University of Pittsburgh Medical Center, Pittsburgh, PA, United States; Meta Reality Labs, Menlo Park, CA, United States; Department of Electrical and Systems Engineering, Washington University in St. Louis, St. Louis, MO, United States; Department of Biomedical Engineering, Washington University in St. Louis, St. Louis, MO, United States; Division of Biological and Biomedical Sciences, Washington University in St. Louis, St. Louis, MO, United States

**Keywords:** diffuse optical tomography, brain mapping, naturalistic stimulus, neural decoding, functional near-infrared spectroscopy

## Abstract

Naturalistic neuroimaging tasks, such as watching movies, are becoming increasingly popular due to being more engaging than resting-state paradigms and more ecologically valid than isolated block-design tasks. As these tasks push the boundaries of naturalistic paradigms, the need for an equally naturalistic imaging device increases. Optical imaging with functional near-infrared spectroscopy (fNIRS) offers a wearable, non-invasive neuroimaging approach. Advancements in high-density diffuse optical tomography (HD-DOT) use a dense array of optical elements to provide overlapping multi-distance fNIRS light measurements for fidelity comparable with functional magnetic resonance imaging (fMRI). Here, to further improve image quality, we increased the density of the imaging grid to 9.75 mm, first nearest neighbor spacing between sources and detectors, leading to a 4-fold increase in measurement density. This very high-density DOT (VHD-DOT) system uses 255 sources and 252 detectors to improve image quality while expanding the field of view. From simulations, the increased density led to improved image resolution across multiple metrics compared with HD-DOT. In vivo group-averaged functional localizer maps are in strong agreement with those collected in MRI on the same cohort of adult participants, indicating that VHD-DOT can be used as a surrogate for fMRI in task-based studies. For a naturalistic movie-viewing task, feature regressor analysis was employed to map audiovisual features from the clip, which also revealed excellent agreement between VHD-DOT and fMRI. Template-based decoding of task and movie-viewing data demonstrates that VHD-DOT signals are repeatable and discriminable, which is necessary for more advanced naturalistic task analyses. This work builds upon previously reported HD-DOT designs to improve the image quality and resolution for whole-head optical imaging. This system is promising for future studies using complex stimuli and analysis protocols, such as decoding, and future work developing wireless VHD-DOT systems.

## 1 Introduction

Functional neuroimaging has enabled the mapping of brain function and revolutionized cognitive neuroscience. Its expanding application in studying health and disease necessitates new, more flexible tools. In particular, the rise of engaging and ecologically valid natural stimuli requires advances in both data collection and analysis protocols (Sonkusare et al., 2019). These tasks are designed to emulate everyday behaviors and provide more realistic insights into brain activity. Naturalistic imaging tasks can include listening (LeBel et al., 2023), movie viewing (Finn & Bandettini, 2021;Vanderwal et al., 2019), reading (Deniz et al., 2019;Kell et al., 2017;Woolnough et al., 2021), or even exploring a virtual reality environment (Bulgarelli et al., 2023;Roberts et al., 2019). Recent studies have even shown naturalistic tasks outperforming resting-state tasks for functional connectivity (Finn & Bandettini, 2021;Gal et al., 2022). For complex functional brain systems, such as the semantic system, naturalistic tasks from visual (Huth et al., 2012) and auditory (Huth, De Heer, et al., 2016;Jain & Huth, 2018) content can provide a diverse generalizable sampling of the feature space. Once encoded or mapped, these tasks can be further employed to decode or predict what a participant saw (Huth, Lee, et al., 2016;Kay et al., 2008;Nishimoto et al., 2011) or heard (Tang et al., 2023) during an imaging session.

While functional magnetic resonance imaging (fMRI) is the gold standard for functional neuroimaging, the technique involves a loud, unnatural scanning environment in the isolated magnet bore. fMRI is also incompatible with patients with implanted electronics or metal, and is often challenging for young children (Raschle et al., 2012). Electroencephalography (EEG) offers portability and a more natural scanning environment while recording the brain’s electrical activity, which has been shown to record reproducible data with naturalistic tasks (Chang et al., 2015;Desai et al., 2021;Haufe et al., 2018;Poulsen et al., 2017). While EEG provides a high temporal resolution, traditional EEG systems offer low spatial resolution, which makes it challenging to resolve different features spatially. However, newly developed high-density EEG arrays can be used to spatially map brain activity (Liu et al., 2017;Marino & Mantini, 2024;Robinson et al., 2017). High-density EEG data are still quite susceptible to artifacts, specifically those generated from eye and muscle movements or other environmental noise, but continued development of data processing tools aims to address these disadvantages. Recent developments in magnetoencephalography with optically pumped magnetometers (OPM-MEG) allow for more naturalistic studies through virtual reality (Roberts et al., 2019), but the requirement for a shielded room limits this imaging system’s capabilities. While each imaging technique has unique advantages and disadvantages, optical imaging methods are especially attractive for naturalistic studies.

Optical imaging with functional near-infrared spectroscopy (fNIRS) offers greater flexibility in imaging compared with alternatives due to being portable, radiation-free, and compatible with implanted metal and electronic devices. fNIRS additionally allows for greater flexibility in imaging due to an open-air scanning environment, no perceivable acoustic noise, and portability (depending on the system), allowing for a more natural scanning environment. Prior fNIRS studies have used virtual reality (Bulgarelli et al., 2023) and in-person interactions (Hirsch et al., 2017) as examples of naturalistic, real-world imaging. However, fNIRS is typically limited by its low spatial resolution. Here, we build upon early developments in high-density diffuse optical tomography (HD-DOT), which used a dense array of optical elements to provide overlapping multi-distance fNIRS light measurements (White & Culver, 2010;Zeff et al., 2007). Together with tomographic reconstruction techniques, these measurements recover brain activations and resting-state networks with fidelity comparable with fMRI (Eggebrecht et al., 2012,^2014^). Recently, HD-DOT has been successful in mapping naturalistic visual (Markow, Tripathy, et al., 2023) and audiovisual stimuli (Fishell et al., 2019;Tripathy et al., 2024). These previous fiber-based HD-DOT systems provide the foundation for wearable HD-DOT systems. Commercial wearable HD-DOT systems provide a completely portable imaging system which have shown success in mapping neural responses in adults (Uchitel et al., 2022;Vidal-Rosas et al., 2021).

With these optical methods, a tradeoff exists between image quality and the number of measurements collected by the imaging system (Wheelock et al., 2019;White & Culver, 2010). Here, a measurement is obtained from a single source–detector pair. Decreasing the spacing between sources and detectors is one approach for increasing the number of measurements. However, several optoelectronic, optomechanical, and computational challenges are associated with these high-density systems. Previously reported HD-DOT systems have addressed many of these challenges, resulting in HD-DOT systems containing approximately 1200–2500 measurements with a typical between-optode spacing of approximately 13 mm (Eggebrecht et al., 2014;Tripathy et al., 2024). While these systems have demonstrated image quality closer to MRI than traditional fNIRS systems, the spatial resolution is still ~13–16 mm. Wearable imaging systems often use tiles containing sources and detectors which can be arranged into a wide variety of arrays. While initial publications used them in isolated regions (Uchitel et al., 2022;Vidal-Rosas et al., 2021), more recently full head coverage has been achieved (Collins-Jones et al., 2024;O’Brien et al., 2024). Recent advances with an ultra-high-density DOT system reported an optode spacing of 6.5 mm, resulting in a spatial resolution of ~9–11 mm (Markow et al., 2025). However, this system had a small field of view covering only the visual cortex.

Here, we highlight our very high-density DOT (VHD-DOT) system with full head coverage, including occipital, temporal, parietal, and frontal areas. The system contains double the number of optodes compared with previous HD-DOT systems and four times more measurement pairs. The 255 sources and 252 detectors are distributed across the head with a spacing of 9.75 mm, resulting in nearly 10,000 total measurements with source–detector separation less than 40 mm. This system was validated through standard functional localizers and naturalistic movie-viewing tasks in eight healthy adult participants. The resulting maps were compared with subject-matched fMRI maps to demonstrate the accuracy of the VHD-DOT imaging system. To highlight the repeatability of the VHD-DOT signals, data were further repurposed to decode localizer and movie stimuli information. The open-air scanning environment and absence of noise provide an improvement over the confined bore of MRI, allowing for increasingly naturalistic experiments, including movie-viewing and in-person interactions. Overall, this work establishes the feasibility of future VHD-DOT imaging studies using naturalistic paradigms such as visual and auditory semantic decoding and provides the fiber-based foundation for future wearable, portable VHD-DOT imaging systems.

## 2 Methods

### 2.1 Simulation analysis

In design of the VHD-DOT system, we first evaluated the effect of the grid density on imaging performance through simulations for systems with nearest-neighbor spacings of 13 mm for HD-DOT (Eggebrecht et al., 2014) and 9.75 mm for VHD-DOT. Here, we simulated single-voxel activations to estimate point spread functions at approximately 200,000 voxel locations within the head across the entire DOT field of view (Markow et al., 2025). Measurements were restricted to only include those with a source–detector separation of less than 40 mm, as greater separations tend to have insufficient signal to noise. To evaluate the image quality for the HD- and VHD-DOT systems, individual point activations were simulated for each voxel within our constrained field of view. The reconstructed images provided analysis of spatially dependent point spread functions (PSF) and were quantitatively assessed as a function of depth to compare the resolution, positional accuracy, and signal to noise (SNR) between the HD- and VHD-DOT imaging systems.

#### 2.1.1 Forward model

To generate the simulated point spread functions, the standard linear forward model for continuous wave DOT systems was employed (Barbour et al., 1991;Durduran et al., 2010;Eggebrecht et al., 2014;Hanli et al., 1995;Markow, Trobaugh, et al., 2023;Wheelock et al., 2019;Zeff et al., 2007). Briefly, the forward model describes how changes in light levels at the surface of the head are related to changes in optical properties within the tissue. This relationship can be modeled linearly, as described inEq. (1).

y=Ax (1)

Here, the vector of measurements from each source–detector pair provides the changes in light levels at the surface (y), for a given differential absorption pattern within the tissue (x). The Jacobian matrix,A, often referred to as the sensitivity matrix, relates these changes at the surface to changes within the tissue and is derived from the Boltzmann Transport Equation (Boas & Yodh, 1997;Maureen et al., 1995). To generate the sensitivity matrix, we built an anatomically based head model using a five-layer segmented head mesh constructed from T1-weighted and T2-weighted MR images from a single participant. This mesh was computed using FreeSurfer (Fischl, 2012) and NeuroDOT (Schroeder et al., 2023) for segmentation, and NIRFAST (Jermyn et al., 2013) for mesh generation. We then position the full array of sources and detectors onto the head model. The diffusion of photons through the mesh was modeled using NIRFAST (Dehghani et al., 2008), which is a finite-element solver.

To make our simulations more realistic, a noise term was included in the forward model to account for measurement noise (Eq. (2)).

y=Ax+n. (2)

Noise was estimated using previously published HD-DOT data (Eggebrecht et al., 2014) and a multivariate Gaussian distribution where variance was dependent on the source–detector separation distance while the covariance between channels was zero. Additional details on the noise model are described inMarkow et al. (2025).

#### 2.1.2 Image reconstruction (inverse model)

Image reconstruction followed established methods for HD-DOT (Eggebrecht et al., 2014;Markow, Trobaugh, et al., 2023;Tripathy et al., 2024;Zeff et al., 2007). To obtain the changes in absorption within the tissue,xfromEq. (1), the sensitivity matrix must be inverted. Here, we use Tikhonov regularization and an additional spatially variant regularization term. The tradeoff between noise and spatial resolution is controlled by the Tikhonov regularization parameter ($\lambda_{1}$) which was set to 0.01 throughout this study based on the literature (Eggebrecht et al., 2014). The spatially variant regularization parameter ($\lambda_{2}$) reduces spatial localization errors induced by Tikhonov regularization biasing images toward the surface of the head. In this study,$\lambda_{2}$is set to 0.1 based on previous studies (Eggebrecht et al., 2014). Additional information regarding image reconstruction is detailed inSupplementary Methods.

#### 2.1.3 Simulation image quality metrics

To assess the difference in image quality between the HD and VHD-DOT systems, we computed image quality metrics including spatial resolution, localization error, effective resolution, and signal-to-noise ratio, using the PSFs reconstructed from the simulated point activations. These metrics are analyzed versus depth between 5 mm and 25 mm from the scalp’s surface (Markow et al., 2025). The spatial resolution (full-width at half-maximum of each PSF), visualized on the MNI152 atlas surface, demonstrates the difference between the VHD-DOT and HD-DOT systems. To evaluate localization error, we computed the Euclidean distance between the point activation location and the centroid of the PSF image. Effective resolution combines spatial resolution and localization error into a single spatial uncertainty measure (White & Culver, 2010). This was computed as the diameter of a circle needed to cover the voxels with intensity ≥50% of the PSF’s maximum from the center of the initial perturbation point. This region was also used to compute the signal-to-noise ratio by dividing the average signal by the standard deviation of the simulated noise as defined inSection 2.1.1within this region. These metrics provide a foundation for the image quality improvements we expect between VHD- and HD-DOT.

### 2.2 VHD-DOT system configuration

This study aimed to develop the largest channel-count DOT system to date, to provide nearly whole-head coverage and improved image resolution over existing HD-DOT systems. This custom-built DOT system has the following instrumentation, system, and cap design.

#### 2.2.1 VHD-DOT instrumentation and design

This system contains 255 laser sources (HL8338MG and HL6750MG, Thor Labs) for illuminating the head at 685 and 830 nm wavelengths with light levels well below the American National Standards Institute’s (ANSI) limits for near-infrared light exposure (1.1 mW/mm^2^, ANSI limit: 4 mW/mm^2^). A dedicated computer with custom software for the illumination patterns controls the light sources. To minimize crosstalk, sources are illuminated with a specific frequency, temporal, and spatial encoding pattern. To extend the system’s dynamic range, a two-pass encoding pattern allows for adequate light collection from nearby (first-nearest neighbor, ~9.75 mm separation) and farther-away detectors (fifth nearest neighbor, ~40 mm separation) (Markow, Trobaugh, et al., 2023;Tripathy et al., 2024). This scheme results in a frame rate of 7.8 Hz. Additional encoding pattern information is given inSupplementary Methods.

Scattered light through the tissue is measured with 252 avalanche photodiode (APD) detectors (C12703-112, Hamamatsu). Signals from each APD are digitized using analog-to-digital converters (ADC) at 96 kHz (Focusrite RedNet A16R MkII). Two computers with custom software display real-time light and noise levels for the digitized signals and are used for recording during data collection.

Light is transported between the sources, detectors, and the participant’s head using fiber optic cables (50-4507-REV1 and 50-4506-REV1, US Fiberoptec Technologies). To avoid any system weight on the participant, fiber weight was supported by a metal frame and a pair of concentric wooden halos. Additionally, to ensure minimal pressure on the top of the head, the motor pad is suspended using a counterbalance and calibrated to ensure the weight does not exceeding approximately 0.5 pounds. The sources, APDs, and ADCs were mounted on aluminum carts (custom design, MiniTec). A system configuration diagram is included inSupplementary Figure S1, along with additional details regarding the instrumentation and design inSupplementary Methods.

Stimulus delivery to the participant was regulated through a fourth computer with the Psychophysics Toolbox 3 package for MATLAB (Brainard, 1997). A display monitor and two speakers were placed in front of the participant and connected to the stimulus computer. To ensure precise timing of our stimulus with the ADC signal outputs, synchronizing signals were sent from the source and stimulus computers to the last two channels of the ASD inputs.

#### 2.2.2 VHD-DOT cap design

To ensure an optimal cap fit across multiple participants, the cap shape was iterated over various designs and based on a representative MRI structural scan from a single participant. Prior HD-DOT caps were constructed manually by molding thermoplastic to a model head and drilling holes for the fiber housing (Eggebrecht et al., 2014;Tripathy et al., 2024). To improve the precision of the cap build, a 3D printing approach was implemented for a more consistent cap construction. The cap was designed in Autodesk Fusion 360 with 6.25 mm diameter holes for the fiber housing spaced 9.75 mm apart on a uniform grid (Supplementary Fig. S2A–D). The cap was 3D printed using TPU 70-A on an SLS printer by Protolabs. To increase cap flexibility for variations in head curvature and size, the cap was separated into four panels to cover the left lateral, right lateral, dorsal, and posterior areas of the head. The lateral and posterior panels were stitched together to form the central band of the cap, which wrapped around the participant’s head, while the dorsal panel remained separate. Previous HD-DOT caps employed hook-and-loop fastener straps to secure the cap onto the participant’s head, often resulting in increased pressure on the forehead and temple. Additionally, individual optodes can cause increased localized pressure on the head, leading to participant discomfort by nonconforming to the head shape or protruding unevenly from the cap. To distribute pressure evenly across the head, a ratchet mechanism was used to tighten the main section of the cap onto the participant’s head, similar to a bike helmet (Supplementary Fig. S2E). Using this technique, we can fine-tune the pressure of the cap to ensure participant comfort during our scanning sessions. To further ensure participant comfort, optical fiber tips were housed in a spring-loaded casing, allowing the cap to conform to the participant’s unique head shape (Supplementary Fig. S2F). The spring-loaded design allows the fiber tip to travel 8 mm, providing sufficient distance to conform to head sizes while reducing the pressure of the optodes on the scalp. These improvements over older designs allow the VHD-DOT cap to maximize the optode-to-scalp coupling and participant comfort necessary for high-quality DOT data.

### 2.3 In vivo human imaging experiments

Data were collected across three imaging sessions for each participant on different days. Two VHD-DOT imaging sessions included functional localizers and movie-viewing tasks, while one MRI session involved collecting the same functional tasks and anatomical images for light modeling. Eleven participants (21–36 years old, 6 male) were scanned for the study, with 1 subject excluded due to insufficient MRI data and 2 excluded for having fewer than 80% of measurements retained after VHD-DOT preprocessing (Supplementary Fig. S3). Eight participants (24–31 years old, 4 male) are included in all subsequent analyses. Informed consent was obtained from all participants, and consent procedures followed the IRB protocol approved by the Human Research Protection Office at Washington University School of Medicine.

#### 2.3.1 Stimulus protocol

The following stimulus protocols were used in both the VHD-DOT and fMRI experiments.Supplementary Table S1lists the tasks completed by each participant included in the primary analysis for this study. Participants completed six reductive functional localizer tasks: (1) auditory, (2) language, (3) visual left, (4) visual right, (5) motor left, and (6) motor right. A movie-viewing task was included as a separate naturalistic neuroimaging task.

##### 2.3.1.1 Auditory stimulation

Participants were asked to maintain central visual fixation on a crosshair and passively listen while spoken word lists were presented at a rate of 1 word per second for 6, 15-second blocks with 15 seconds of silence between blocks (Eggebrecht et al., 2014).

##### 2.3.1.2 Language stimulation

Participants were asked to maintain central visual fixation while nouns were presented at one word per second. Participants were instructed to imagine themselves saying a corresponding verb aloud for each word. Words were presented for 15 seconds per block, followed by 15 seconds of rest for a total of 6 blocks (Eggebrecht et al., 2014).

##### 2.3.1.3 Visual stimulation—left and right

Participants were asked to maintain central fixation on a crosshair while black-and-white wedge checkerboards were flickered at 8 Hz in either the bottom left or right quadrant of the screen for 10 seconds, followed by 24 seconds of rest. A total of 16 blocks (8 blocks per side) were shown to the participant in a pseudorandomized order for a single run. This task contained two conditions.

##### 2.3.1.4 Motor stimulation—left and right

Participants were asked to remain as still as possible and maintain visual fixation on either a centrally presented crosshair, the letter “L”, or the letter “R”. While the “L” was presented on the screen, participants were instructed to tap the fingers of their left hand against their left thumb at a rate of 2 Hz. Similarly, for the letter “R”, participants were asked to tap their right fingers against their right thumb. These letters were presented for a duration of 10 seconds, with 24 seconds of rest following each block. Blocks were presented in a pseudorandomized order for a total of 16 blocks (8 blocks per hand). This task contained two conditions.

##### 2.3.1.5 Movie viewing

For movie-viewing tasks, participants were asked to watch a 10-minute audiovisual clip from The Good, The Bad, and The Ugly without central fixation (Fishell et al., 2019;Hasson et al., 2004). This task is intended to emulate a natural movie-viewing environment. Participants viewed the movie clip twice in a single imaging session to allow for repeatability analysis.

#### 2.3.2 MRI data collection and processing

MRI data were collected for subject-specific head modeling and a subject-matched comparison with the VHD-DOT data. The MRI data were collected on a separate day following VHD-DOT data collection. MRI scans were conducted on a Siemens Magnetom PRISMA Fit 3.0 T scanner, with an iPAT-compatible 20-channel head coil. Anatomical T1-weighted MPRAGE (echo time (TE) = 3.13 ms, repetition time (TR) = 2,400 ms, flip angle = 8°, 1 × 1 × 1 mm isotropic voxels) and T2-weighted (TE = 84 ms, flip angle = 120°, 1 × 1 × 1 mm voxels) scans were collected for each participant at the beginning of each MRI scan. Functional images were then collected using a series of asymmetric gradient spin-echo echo-planar (EPI) sequences (TE = 33 ms, TR = 1,230 ms, flip angle = 63°, 2.4 x 2.4 x 2.4 mm isotropic voxels, multi-band factor = 4) to measure the blood oxygenation level-dependent (BOLD) contrast.

Data were preprocessed using fMRIPrep 22.0.2 (Esteban, Blair, et al., 2018;Esteban, Markiewicz, et al., 2018), which is based on Nipype 1.8.5 (Gorgolewski et al., 2011,^2018^). More information on the preprocessing methods is given inSupplementary Methods. To match the VHD-DOT preprocessing, data were detrended and bandpass filtered (0.02–0.2 Hz) before smoothing with an isotropic Gaussian smoothing kernel (10 mm FWHM). Data were converted to a percent BOLD change measurement by subtracting and dividing by the average BOLD signal in each voxel over time (Markow et al., 2025).

#### 2.3.3 VHD-DOT data collection

The VHD-DOT cap fit procedure is a crucial first step in data collection to ensure the maximum number of optodes are in contact with the participant’s scalp comfortably and consistently across scan sessions. Our cap fit procedure follows previously described protocols with some modifications due to improvements in our VHD-DOT cap (Eggebrecht et al., 2014;Tripathy et al., 2024). Long-haired participants start by having their hair parted down the middle and across the back of the head to form two ponytails on the left and right sides of the head. The participants were then asked to sit comfortably in the imaging chair and place their head in the cap. The chair height is then raised or lowered so the cap rests near the participant’s ears. The participants are then handed two straps to comb the optodes through their hair and ensure coupling onto the scalp. Fiducial markers on the cap are aligned to the tragus on each side of the head for cap placement consistency and symmetry. The front strap mechanism is tightened onto the head using a ratchet similar to those on a bike helmet for an even distribution of pressure across the head. The top section of the cap that rests on the participant’s head is then lowered and combed through the participant’s hair before being attached to the side panels of the cap using a lacing system to ensure an even distribution of pressure across the participant’s head. Cap fit is assessed in real time using data quality visualizations from laboratory-built software, as well as feedback from the participant. As with other fNIRS/DOT systems, performing a good cap fit with VHD-DOT is a skilled activity requiring training and balancing several goals, mostly data quality and subject comfort. Key to performing the cap fit is the use of real-time visualization that maps data quality across the entire cap. These are used to identify areas of the cap with poor optode-to-scalp coupling. Generally, with large arrays, it is important to emphasize global fitting maneuvers, like side-to-side, back-to-front, or up-down combing actions, rather than focusing on individual optodes (with 512 optodes, single fiber optimization is not practical). Once global combing actions and cap tightening have been optimized, individual optodes were adjusted to comb through hair and improve contact with the scalp based on the real-time data visualizations. However, given the large number of optodes, individual optode optimization was typically limited to <15 optodes. This process was continued until the visualizations exhibited our expected optode-to-scalp coupling based on experience or until the cap fit procedure lasted more than 25 minutes. This procedure takes approximately 15 minutes for optimal cap placement with a well-trained scan team. Once completed, photographs of the cap on the participant’s head are recorded with a camera from at least seven angles.

### 2.4 VHD-DOT data processing

#### 2.4.1 VHD-DOT preprocessing

DOT data were processed similarly to previously reported studies (Eggebrecht et al., 2014;Markow, Trobaugh, et al., 2023;Tripathy et al., 2024). Raw light levels from the two detector computers are first combined based on initial synch pulses generated from the stimulus and converted to differential log-mean ratios across time. For an initial assessment, visualizations of raw data quality are generated, including the light fall-off curve to visualize the mean light level as a function of source–detector distances and the root mean square (RMS) averaged power spectra to visualize the cardiac pulse peak. Noisy channels are rejected if the temporal standard deviation across the run is greater than 7.5%, and participants are excluded if less than 80% of measurements across all runs are retained. Data are detrended and highpass filtered using a 0.02 Hz cutoff, and lowpass filtered with a 1 Hz cutoff. Superficial signal regression is applied to remove scalp-based global systemic signals from the data by averaging the first nearest-neighbor measurements and regressing them from each measurement (Gregg et al., 2010;Zeff et al., 2007). Data are lowpass filtered again with a cutoff of 0.2 Hz, and downsampled to 1 Hz for further analysis.

#### 2.4.2 Image reconstruction and spectroscopy

To account for variations in head tissue structure, subject-specific light models were constructed for each participant using their anatomical MRI data. The following process is repeated for each subject in order to obtain subject-specific light models. Here, we use a two-fold approach by adjusting the grid location using photographs from the imaging session and comparisons with subject-specific fMRI data from a word hearing task. First, a grid of sources and detectors is positioned on an MRI-generated head mesh using anatomical landmarks, such as the tragus and inion, and photographs collected during VHD-DOT data collection using a standard phone camera. The grid of optodes is adjusted through x, y, and z translations and rotations to move the optodes around the anatomical mesh. Once a light model is generated from the optode positions, we perform a second step using functional localizers focusing on the word hearing maps reconstructed from the subject-specific head model and the subject-specific fMRI data. To compare the word hearing maps, the Dice coefficient is computed by first thresholding and binarizing the fMRI and VHD-DOT maps at 20% of the maximum beta value for each map. If the Dice coefficient is greater than 0.2, the subject-specific light model is considered complete. We use analytical estimates to generate position updates (positional errors between the two responses). We keep iterating until the dice coefficient is >0.2, or a maximum of 5 iterations, whichever occurs first (Supplementary Fig. S4). Due to the differences in head shape, the number of measurements varies slightly between participants in the range of 9,408 to 10,330 total measurements for source–detector distances less than 40 mm. These subject-specific light models are used to process each subject’s VHD-DOT data in the study using the previously described image reconstruction inverse problem. Since these images represent differential absorption for each time point, we use spectral decomposition to obtain relative changes in oxygenated and deoxygenated hemoglobin concentrations. InEq. (3), the vectorΔCcontains the oxy- and deoxy-hemoglobin concentrations, whileEis the extinction coefficient matrix (Markow, Trobaugh, et al., 2023;Tripathy et al., 2021).

$$\Delta \text{C}=E^{-1}x.$$ (3)

#### 2.4.3 Data quality metrics

Several metrics are used to assess real-time data quality across each imaging run to evaluate individual sessions and participants (Eggebrecht et al., 2014;Tripathy et al., 2024). The light intensity plotted as a function of source–detector separation distance, also referred to as the light-fall off curve, captures key information such as the dynamic range and spread of the light measurements across the first through fifth nearest neighbors. Due to the large number of measurements from the VHD-DOT system, the light fall-off is plotted by cap section, allowing for individual assessment across regions, and combined across the system (Supplementary Fig. S5). Since data with a temporal variance greater than 7.5% are excluded in preprocessing, a histogram is used to visualize the proportion of measurements that are being retained. With the goal of measuring blood oxygenation, both the Fourier spectrum and a 20-second block of measurements are visualized to evaluate the pulse. To assess the cap placement with respect to pulse, the pulse signal to noise across the cap is computed as a ratio of signal power in the 0.5–2 Hz frequency band to the bandwidth-scaled median power in flanking frequency ranges across the cap. Together, these data quality metrics are tools to assess each imaging session to ensure sufficient data quality for cortical mapping.

#### 2.4.4 Analysis of functional localizer stimuli

To generate task maps from the functional localizer tasks, a general linear model (GLM) was constructed for each task run to compute the stimulus–response beta values relative to the rest blocks. The hemodynamic response function used for our analysis was previously computed using HD-DOT data in adults (Hassanpour et al., 2014). Contrast maps between the left- and right-sided conditions were computed for the motor and visual lateralized stimuli. Beta maps were averaged across all runs for each individual subject and then affine transformed from subject-specific space to the MNI152 atlas space. Group-level maps were generated by computing the fixed effect t-statistics across each subject’s averaged task response following fNIRS and DOT best practices (Yucel et al., 2021). Maps were plotted on the MNI152 atlas surface meshes. To ensure an equivalent comparison between VHD-DOT and MRI, functional localizer task data were similarly processed using the fMRI BOLD responses. VHD-DOT and MRI group-level maps were binarized based on the maximum T-statistic at both 25% and 50% of the maximum value. These binary maps were used to plot overlap maps and compute the Dice coefficient as an overlap metric. To evaluate the oxy- and deoxyhemoglobin responses for each task, group-level time trace plots were computed through block averaging each task within a region of interest. These regions of interest were generated by thresholding the group-level fixed effect t-statistic maps at 50% of the maximum value (Supplementary Fig. S6). Oxy-, deoxy-, and total hemoglobin responses within each block were averaged across all voxels within the region of interest and then averaged across runs. The mean time traces were plotted with the standard error across the blocks for each task.

#### 2.4.5 Neural decoding of functional localizer stimuli

To highlight the reproducibility of the VHD-DOT signals, a spatiotemporal template-matching approach was adapted for six-way functional localizer stimuli identification based on prior studies (Markow, Tripathy, et al., 2023;Tripathy et al., 2021). For each subject, a single run of each localizer task was selected within the same imaging session for a total of 6, 30-second blocks for each task. Since the motor and visual tasks contained 8, 34-second blocks, these tasks were constrained to the first 6 blocks from each run, with each block truncated at 30 seconds. For temporal feature selection, the first 4 seconds of each block were removed to avoid transient stimulus onset responses, and response duration was limited to 16 seconds to capture the hemodynamic response (Tripathy et al., 2021). Data for each task were divided by interleaving blocks into training (three blocks) and testing (three blocks) datasets. The training data were averaged into a spatiotemporal (voxel x time) template for each task for a total of six templates. The test data consisted of 18 blocks (3 blocks from each of 6 tasks). A representative illustration of the template and test trial design is included inSupplementary Figure S7.

For our template-matching classification approach, the spatial Pearson correlation was computed between each average template block and the single trial test data blocks at each time point. Additionally, a brain mask was generated for each subject from the anatomical MRI data to restrict the voxels for decoding to only those within the brain and neighboring cerebrospinal fluid. Using a maximum likelihood approach, the template with the highest correlation with the trial response determined the decoding output. For each subject, a confusion matrix was generated by tracking the number of times each trial clip was decoded across the total number of trials. Decoding performance was computed as the total percentage of correctly decoded trials. To assess significance, decoding performance was compared with chance, that is, 1/(total number of templates). Decoding results were aggregated across subjects by summing over the subject-level confusion matrices and computing a single decoding performance value across all decoding trials. This decoding strategy was performed on oxy- and deoxyhemoglobin VHD-DOT responses and the fMRI BOLD data. The fMRI BOLD data were constrained to the VHD-DOT field of view for appropriate comparisons.

#### 2.4.6 Mapping of a naturalistic audiovisual movie clip

We selected two approaches for the mapping of the audiovisual movie clip responses. To assess repeatability, Pearson pairwise correlations were computed across all voxels within the VHD-DOT field of view between each viewing of the clip for both the VHD-DOT and fMRI responses. These spatiotemporal correlation maps were affine transformed to the MNI152 atlas space and group-level mapped by computing the fixed effects t-statistics across all subjects, similarly to the functional localizer group mapping. This pairwise correlation approach highlights the brain regions consistent across multiple viewings of the same movie clip and further establishes the repeatability of our VHD-DOT signals compared with fMRI BOLD responses.

To capture some of the numerous features within the audiovisual movie stimulus, regressors were constructed to represent the clip’s low- and high-level auditory and visual features. These features included the auditory envelope and luminance as low-level auditory and visual components, while high-level features such as speech, faces, and hands were selected to capture the clip’s dynamics. More details on these features and their construction are given inFishell et al. (2019). Briefly, we computed luminance by averaging the pixel intensity for each frame after converting it to grayscale. To track variations in audio intensity, we calculated the auditory envelope by averaging the logarithmic power modulations across 25 frequency bands, creating a single time course for the movie clip. Three raters manually coded high-level features (speech, faces, hands) in 1-second bins, recording a binary rating for the presence or absence of each feature. We resolved discrepancies between raters through re-evaluation to reach a consensus. After convolving each raw feature time trace with a canonical hemodynamic response function, we bandpass filtered the traces to match the VHD-DOT preprocessing parameters. Finally, we computed the temporal correlation between each feature regressor and the measured cortical response for each voxel within the VHD-DOT field of view. Group-level maps were generated by computing the fixed effects t-statistics across all subject spatial maps for each audiovisual feature.

#### 2.4.7 Neural decoding of audiovisual movie clip

To further establish the reproducibility of VHD-DOT signals, we used a spatiotemporal template-matching approach to identify which subset of the movie clip the participants viewed at a given time. This approach follows the functional localizer decoding, with the first viewing of the movie as our template and the second viewing of the movie as test data. Given the naturalistic audiovisual qualities of the movie-viewing task, this decoding paradigm offers preliminary insight into more complex decoding study designs. Further, given the long duration of our movie clip (10 minutes), we systematically divided the movie clip to provide a more challenging decoding problem using more templates and a shorter duration of neural activity. We divided the movie into 2, 4, 8, 15, and 30 clips with durations of 300, 150, 75, 40, and 20 seconds, respectively, separating each segment by 6 seconds to avoid transient stimulus responses. We again used a template-matching approach constrained to the brain mask, applying maximum spatial Pearson correlations across time between the template and test data as our decoder. Using the maximum correlation between the test movie and the templates, we predicted which clip the participant was viewing based on their neural activity. We tracked decoding performance using a confusion matrix for each subject individually and aggregated it across the group for each number of template options. Decoding performance was computed as the ratio of trials that were correctly decoded, with the chance level determined as 1/(total number of templates) for oxy- and deoxyhemoglobin in VHD-DOT and BOLD in fMRI.

## 3 Results

### 3.1 System design potential via simulation

**Fig. 1.**
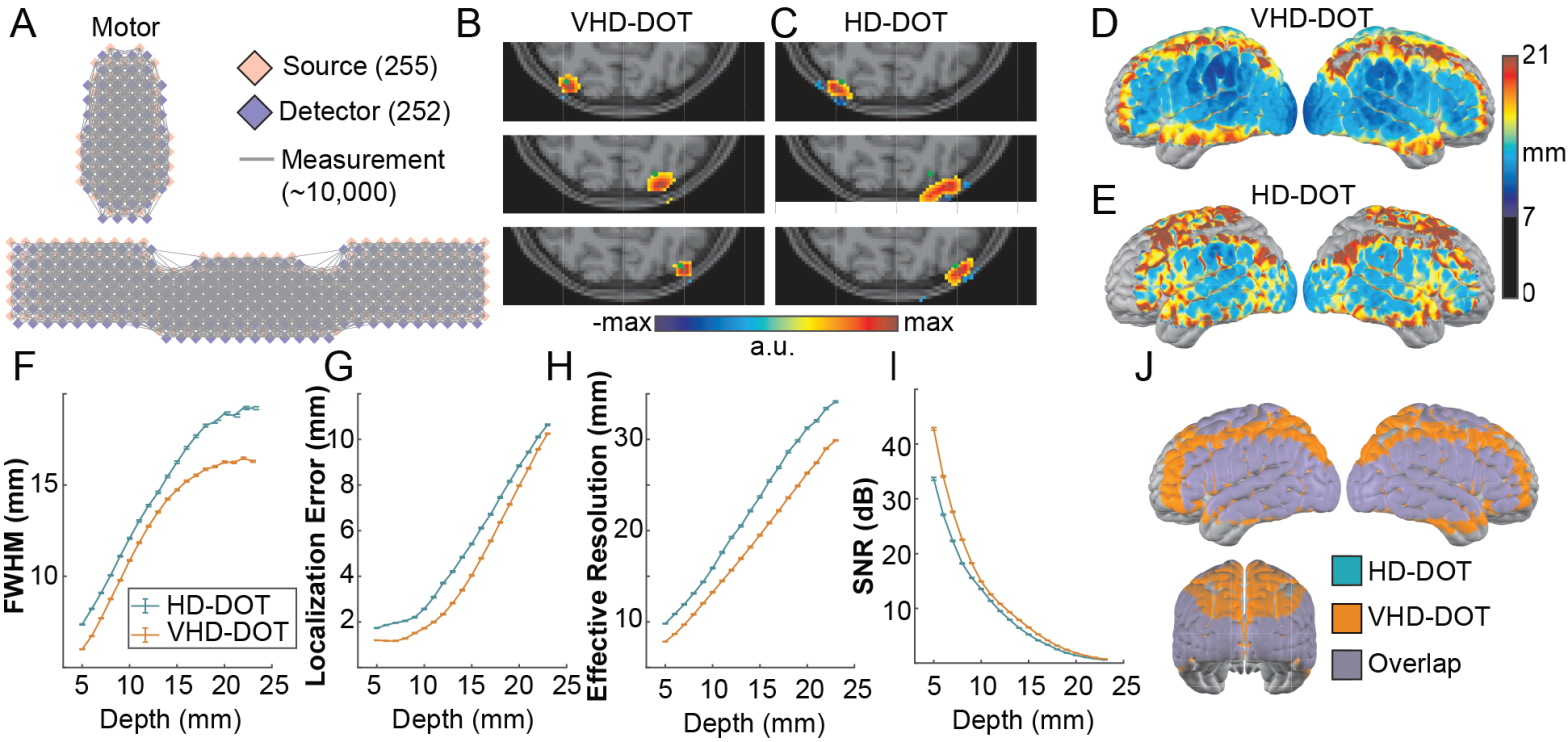
Simulation of VHD-DOT versus HD-DOT: VHD-DOT samples approximately 9,000–10,000 measurements using 255 sources and 252 detectors distributed across the head with a separation of 9.75 mm (A). Point spread functions were reconstructed using the VHD-DOT grid (B) and an HD-DOT grid (C) to evaluate the image quality improvements between the imaging systems. The full width at half maximum of the point spread functions was computed and plotted on the MNI152 atlas (D, E) to evaluate the image resolution improvements for the VHD-DOT system. Image quality metrics, including FWHM (F), localization error (G), effective resolution (H), and SNR (I), were subsequently computed to highlight the improved image quality of VHD-DOT over traditional HD-DOT systems. The increase in the overall number of sources and detectors contributed to a larger field of view for the VHD-DOT system in comparison with the HD-DOT system (J), with double the number of cortical voxels.

The VHD-DOT imaging system (with 255 sources and 252 detectors) doubled the number of sources and detectors of previously established HD-DOT systems (Eggebrecht et al., 2014;Tripathy et al., 2024). This results in approximately 9,757 measurements (<40 mm source–detector separation) across both wavelengths (Fig. 1A) for VHD-DOT. Compared with an equivalent full-head HD-DOT, this increase of 4x in the number of measurements is expected to improve the image quality for the VHD-DOT system. Simulated point spread functions were generated from the VHD-DOT and HD-DOT grids to quantify and evaluate these potential improvements. Single point targets (green dots inFig. 1B, C) were reconstructed for VHD-DOT (Fig. 1B) and HD-DOT (Fig. 1C) and plotted in volume space. From these reconstructed point targets, we found that the point spread function for the VHD system is more localized and better resembles our target than the HD system. Visualizing the full width at half maximum of all simulated point spread functions within the field of view highlights the spatial resolution improvement between the VHD and HD-DOT imaging systems (Fig. 1D, E). Generally, the PSFs for both systems are best in the middle of each section of the imaging array, with lower performance toward the edges of the sections. Overall, VHD-DOT provides consistently higher performance. This highlights the direct improvement in image resolution from the VHD-DOT system (FWHM 10–13 mm) compared with the HD-DOT system (FWHM 13–16 mm). Image quality was further assessed by plotting the FWHM of the point spread functions, localization error, effective resolution, and signal-to-noise ratio, each as a function of depth (Fig. 1F–I). Across all four metrics, VHD-DOT outperforms HD-DOT, further indicating that the 9.75 mm between-optode spacing improves image resolution.

The large optode count of the VHD-DOT system allowed for further expansion of the field of view to achieve nearly whole-head coverage. Compared with the field of view of our previous HD-DOT system (Fig. 1J), we see a substantial increase with additional frontal and parietal cortex coverage. Aside from the increased channel count, the VHD-DOT field-of-view samples approximately double the number of cortical voxels compared with the HD-DOT imaging system. Compared with the full coverage of MRI, VHD-DOT captures approximately 36.9 ± 0.05% of cortical voxels. This larger field of view allows for more complex imaging studies that require whole-head imaging, such as the mapping of semantic content across the cortex (Huth, De Heer, et al., 2016) or precision functional connectivity studies (Gordon et al., 2017).

### 3.2 System characterization

**Fig. 2.**
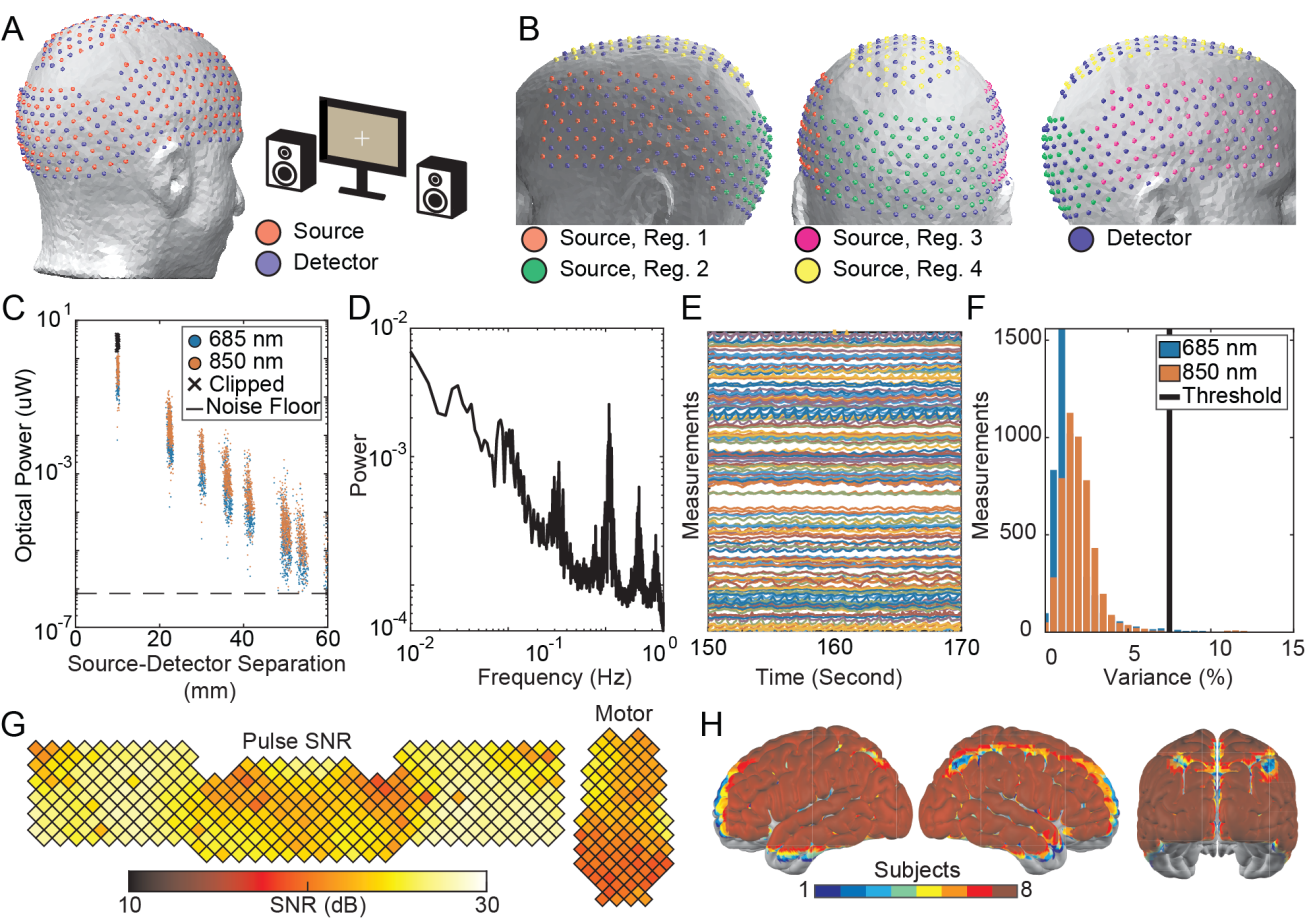
VHD-DOT system characterization: Sources and detectors were distributed across the head (A) with sub-regions of the cap containing 64 sources each (B) for our spatial encoding scheme. Representative data quality figures from individual subjects include the light fall-off for the left panel of the cap (C), which visualizes the optical power as a function of source–detector separation, the Fourier spectra (D), which highlights the pulse peak around 1 Hz, measurement time traces (E) additionally for visualizing the pulse, and the measurement histogram (F) indicating the number of measurements below our 7.5% variance threshold. The mean bandlimited pulse SNR (G) provides further insight into the SNR across the cap, which is used to assess the cap fit. The unique field of view for each subject’s individual light model highlights the variability in the cap coverage based on the size and shape of the participant’s head (H). However, the cap still covers much of the same regions across each participant.

Based on the image quality and field-of-view improvements for the simulated VHD-DOT system, the imaging system was constructed. Here, the 255 sources and 252 detectors are arranged in a grid pattern across the head with a closest between-optode spacing of 9.75 mm (Fig. 2A). To ensure a high temporal resolution with minimal crosstalk, four source encoding regions are used across the cap (Fig. 2B). Validation of the system’s electro-optical elements began with assessing the light falloff from the imaging system during an in vivo imaging session. Due to the large number of measurements, the light falloff plots were divided by cap section (left, right, visual, and dorsal) in addition to a combined plot of all measurements (Supplementary Fig. S5). Here, we show the left panel light falloff (Fig. 2C), which demonstrates a log-linear pattern with measurements well above the noise floor, even at source–detector distances approaching 60 mm. From the first nearest neighbor measurement pairs, we achieve a dynamic range of 10^6^. To initially assess the imaging system with in vivo imaging, we visualized the raw data quality of the system to ensure that we could detect the participant’s pulse, a necessary first step for imaging cerebral oxygenation. The Fourier spectra (Fig. 2D) indicate a clear pulse peak around 1 Hz, which is further verified through the pulsatile shape in the individual time traces (Fig. 2E). Temporal variance in the measurements was captured by visualizing the histogram of measurement variance across both wavelengths (Fig. 2F). The majority of measurements contain variance less than the threshold of 7.5%. Finally, the pulse SNR across the cap (Fig. 2G) further indicates the presence of a pulse signal along with a visualization of the cap fit with lower SNR regions being associated with worse optode-to-scalp coupling. Together, these raw data quality metrics provide a comprehensive overview of the raw data quality for a given imaging session. Given that the cap conforms differently across subjects and each participant’s light model is slightly different based on their head geometry, we overlaid the fields of view for each individual participant to assess variability across subjects. Here, we see that the field of view exhibits modest differences across the group, but overall, the cap is positioned similarly on each participant (Fig. 2F).

### 3.3 System validation via functional localizers

**Fig. 3.**
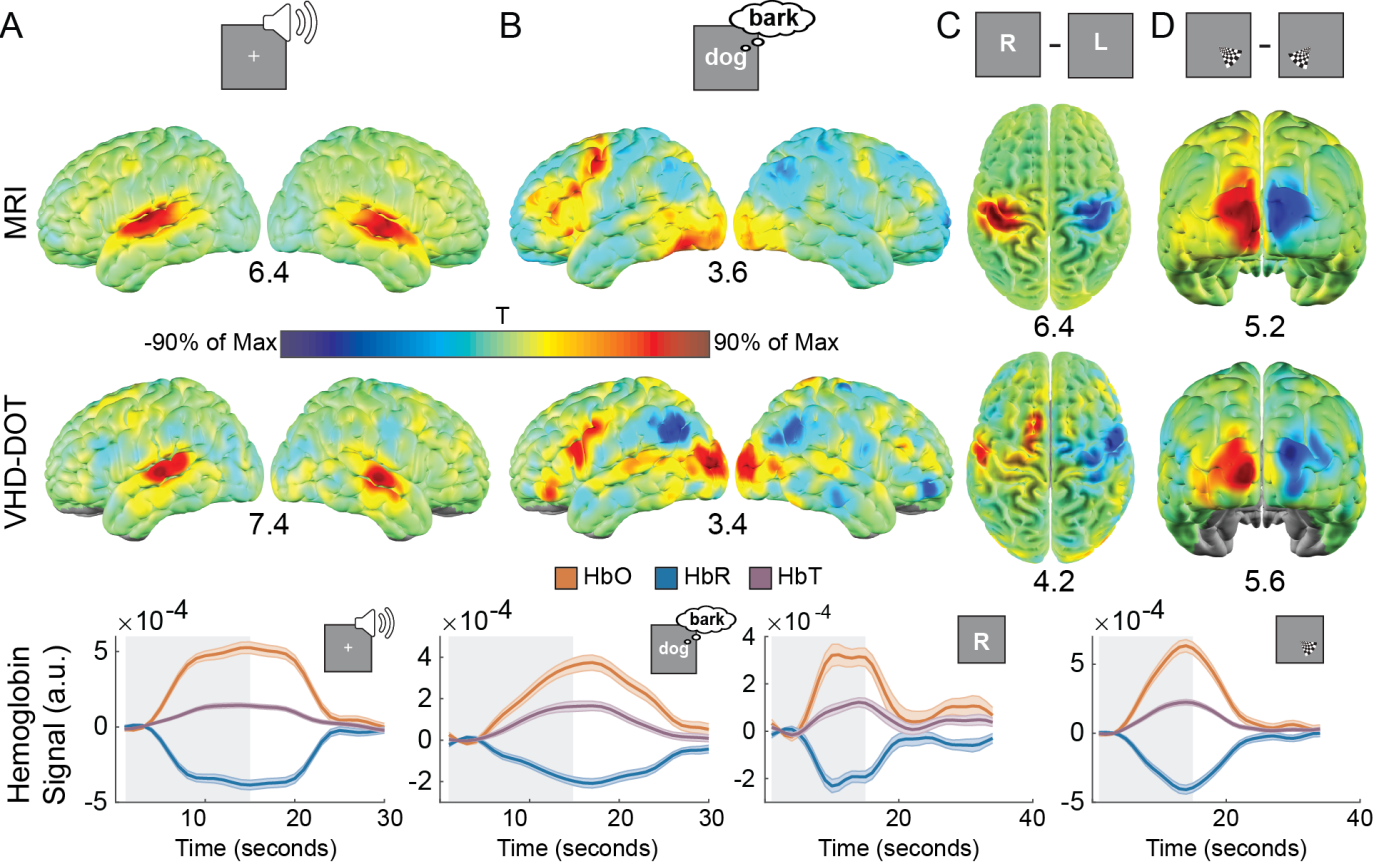
Functional localizer task maps: Fixed effect t-statistic maps were generated for group mapping of the localizer tasks for both VHD-DOT oxyhemoglobin data (middle row) and MRI BOLD data (top row). Tasks included word hearing (A), verb generation (B), finger tapping (C), and flashing checkerboards (D). Values under the maps indicate the maximum t-statistic value in the plot. Maps are visually consistent between the VHD-DOT and MRI data, indicating that the VHD-DOT system successfully maps functional localizer tasks. Group time traces for each task (bottom row) were computed for oxyhemoglobin (red), deoxyhemoglobin (blue), and total hemoglobin (purple) for the VHD-DOT data to visualize the data temporally. Regions in gray indicate the duration of the task.

A battery of functional localizers was selected to validate the system through in vivo experiments. Individual subject activation maps from VHD-DOT indicate that the system can adequately map brain activity across the cortex on a single run level for all tasks (Supplementary Fig. S8). For the block-design word hearing task, similar bilateral regions of the auditory cortex were activated with comparable maximum t-statistic values for both VHD-DOT and fMRI (Fig. 3A). The verb generation task resulted in activations in language areas as well as the visual cortex from the words flashing on the screen (Fig. 3B). Similarly, the contrast maps for both the finger tapping and visual tasks activated contralateral regions of the motor (Fig. 3C) and visual (Fig. 3D) cortex, respectively. To quantitatively compare the VHD-DOT and fMRI results, binarized overlap maps were generated for each task (Supplementary Fig. S9) and the Dice coefficient was computed (Supplementary Table S2). To further visualize the VHD-DOT data temporally, oxy-, deoxy-, and total hemoglobin time traces for each task were averaged over a selected region of interest based on the t-statistic maps (Supplementary Fig. S6). Across all tasks, we see the expected increase in oxygenated hemoglobin and a decrease in deoxygenated hemoglobin during the task blocks indicated with the gray-shaded regions (Fig. 3). This further indicates that the VHD-DOT system can map functional tasks across the cortex.

**Fig. 4.**
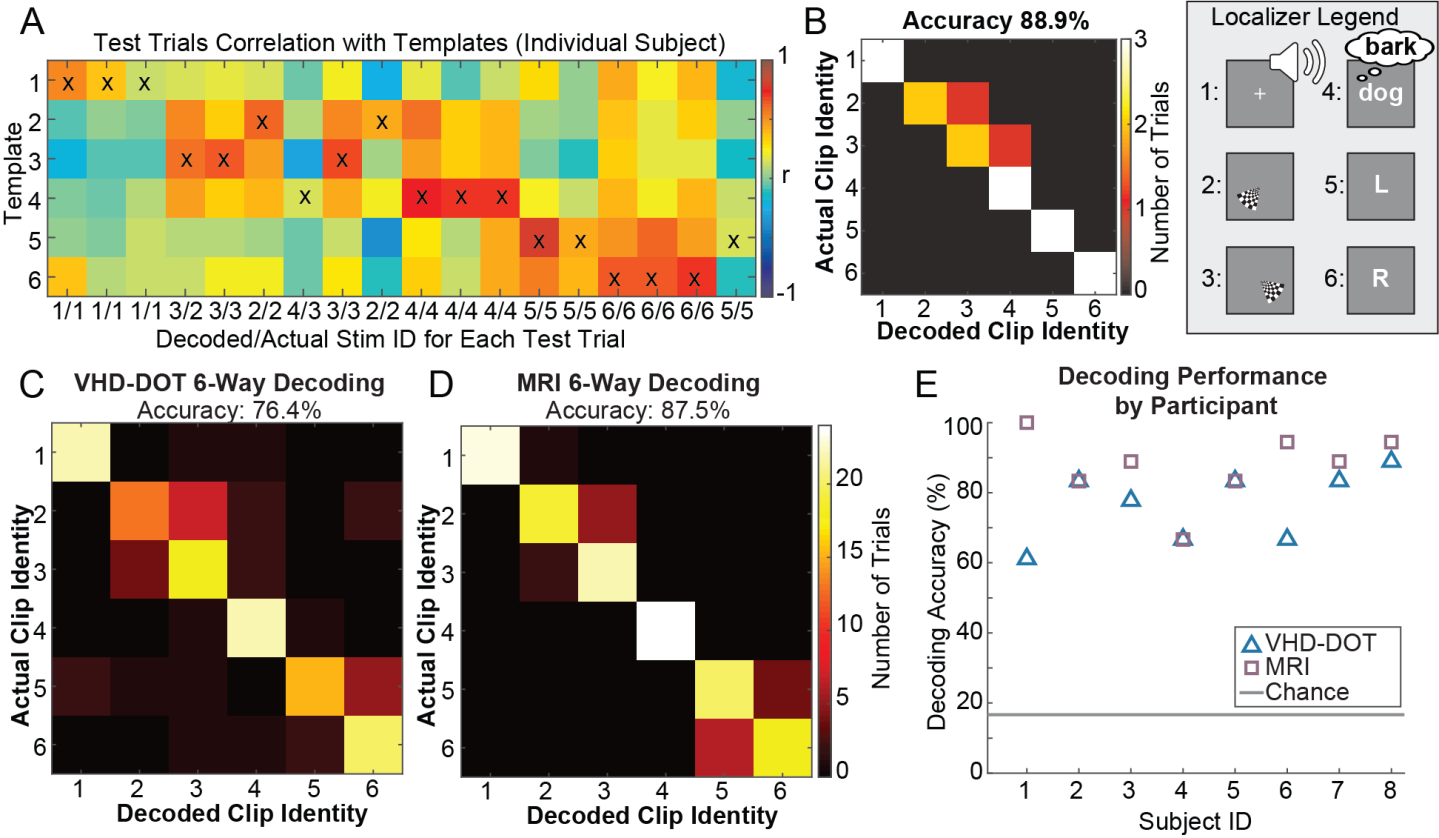
Six-way functional localizer decoding: Spatiotemporal templates were constructed for each functional localizer task (localizer legend) and used for trial-based decoding to evaluate the repeatability of the VHD-DOT signals. Correlations between the template and each trial were computed, and the maximum correlation value (denoted by an x in each column) was used to predict the corresponding localizer task, shown here for a single representative participant (A). The actual versus decoded clip identities were combined in confusion matrices for each participant (B) and aggregated across all participants for both VHD-DOT (C) and MRI BOLD (D). Both the VHD-DOT and MRI BOLD decoding accuracies were well above chance (16.7%) at 76.4% and 87.5%, respectively. Decoding performance was reported individually for each subject (E) to directly compare the VHD-DOT and MRI BOLD results for each participant.

To validate the repeatability and discriminability of our VHD-DOT signals, we took a spatiotemporal template-matching approach to identify which task a participant completed based on a single trial of data. Six blocks were selected for each localizer task (word hearing, verb generation, left/right finger tapping, left/right checkerboard viewing). Pearson pairwise correlations between the trial and each template were computed for each participant (Fig. 4A). The maximum correlation was used to predict which task the participant completed at a given time, with prediction results aggregated in a confusion matrix (Fig. 4B). We computed the overall decoding accuracy for this representative participant to be 88.9%. These confusion matrices were summed across all participants to compute an overall decoding accuracy (Fig. 4C). Here, we achieved a decoding accuracy of 76.4%, which is well above chance (16.7%). We similarly computed the localizer decoding performance using the MRI BOLD data, which achieved an accuracy of 87.5% (Fig. 4D). The MRI decoding performance can be treated as an approximate ceiling for the decoding performance we could achieve with VHD-DOT in these participants. Since data quality and participant compliance can impact decoding performance, VHD-DOT and MRI performance were plotted for each individual subject (Fig. 4E). Overall, all participants achieved decoding performance greater than chance, with three subjects having equivalent VHD-DOT and MRI performance. These results suggest that VHD-DOT data are highly repeatable even at a single trial level across multiple subjects. Functional localizer activation maps and decoding results generated from deoxyhemoglobin data are given inSupplementary Figure S10. We achieve similar results with both oxyhemoglobin and deoxyhemoglobin, demonstrating that our VHD-DOT system is sensitive to multiple chromophores.

### 3.4 System validation via naturalistic stimuli

**Fig. 5.**
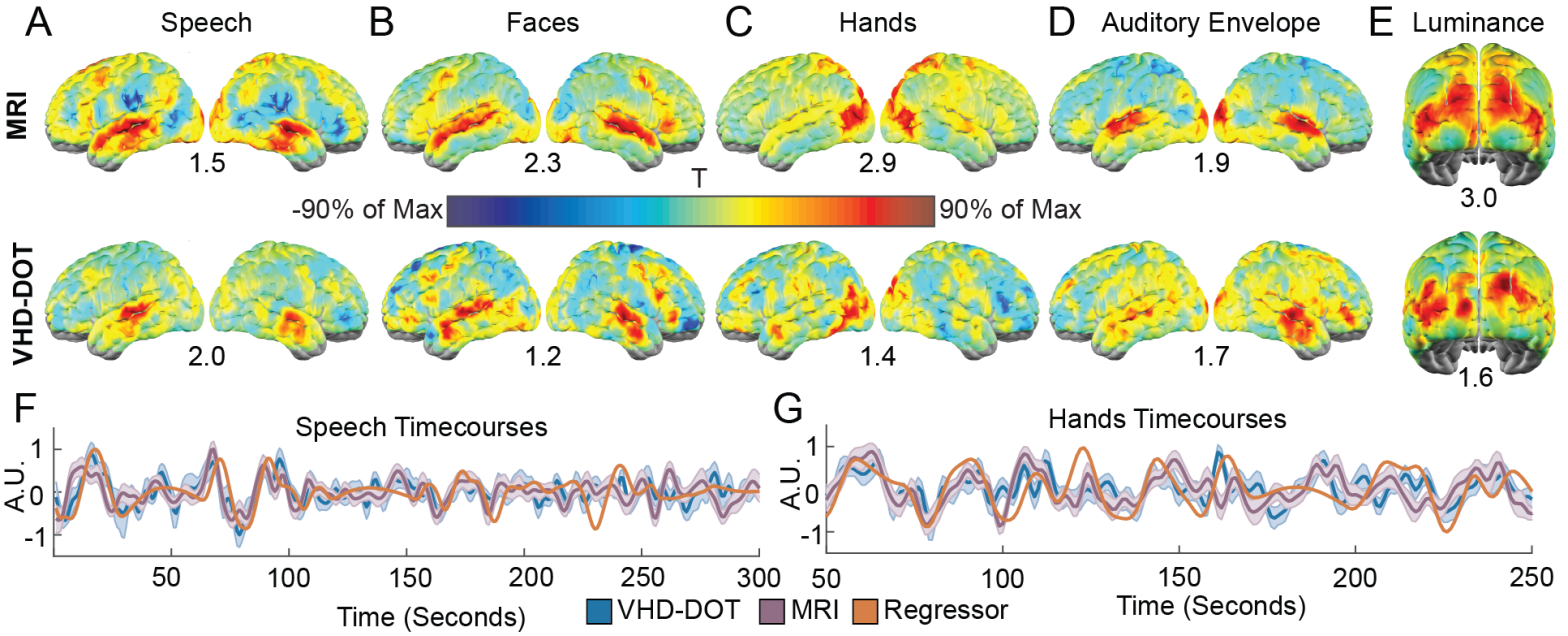
Feature analysis of movie-viewing task: Movie viewing was assessed using feature regressors to map multiple processing pathways in parallel. Individual subject maps were combined using fixed-effect t-statistics with the maximum t-statistic value indicated under each map. High-level features, including speech (A), faces (B), and hands (C), were mapped using VHD-DOT with strong similarity to fMRI. Similarly, low-level features included auditory envelope (D) and luminance (E). These features cover a range of auditory and visual regions of the brain. Group average time traces were plotted for the speech (F) and hands (G) regressors for a single voxel within the auditory and visual cortex, respectively. These time traces indicate the temporal similarity between the voxel hemodynamics and the feature regressor.

As a complex stimulus, movie-viewing tasks can be used to map multiple processing pathways in parallel via feature regressor analysis. Bilateral auditory activations from the speech regressor (Fig. 5A) resemble the auditory activations derived from the word hearing localizer task for both VHD-DOT and MRI (Fig. 3A). Areas associated with face processing, including the superior temporal sulcus and occipital face area, are similarly activated in both the VHD-DOT and MRI data (Fig. 5B). The hands regressors selected for voxels within the visual cortex (Fig. 5C), such as the middle temporal area associated with motion perception. The low-level features primarily activated regions within the auditory and visual cortex for the audio envelope (Fig. 5D) and luminance (Fig. 5E) regressors, respectively. To visualize the similarity between the VHD-DOT and MRI signals with the regressor time traces, the average signal across all movie runs was computed for a single voxel within the auditory (MNI coordinates: [48, 29, 22]) and visual (MNI coordinates: [43, 7, 22]) cortex. The auditory cortex time traces were plotted alongside the speech regressor (Fig. 5F) and the visual cortex time traces with the hand (Fig. 5G) regressor. The Pearson correlation between the hand regressor and VHD-DOT visual time trace was 0.35, while the correlation was 0.37 for the MRI data. Similarly, the speech regressor correlation with an auditory seed was 0.47 for VHD-DOT and 0.20 for MRI.

**Fig. 6.**
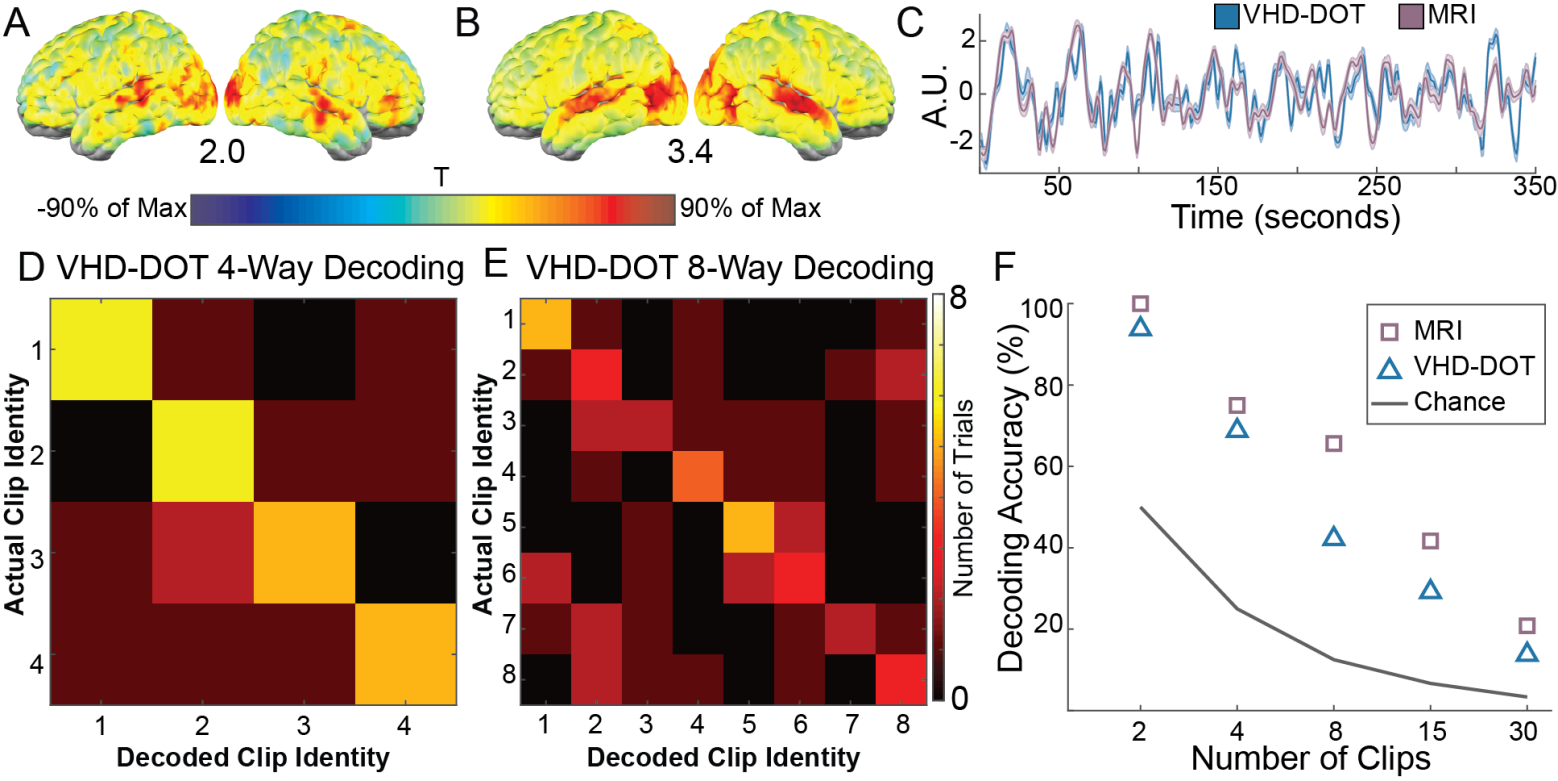
Template-based movie decoding: Movie viewing drives repeatable brain activity between viewings of the same clip. Spatiotemporal correlations were computed between the first and second movie viewing runs for all participants in VHD-DOT (A) and MRI (B), and were group averaged using fixed effects t-statistics. The maximum t-statistic value is indicated under each map. A representative oxyhemoglobin time trace for VHD-DOT and MRI appears highly correlated with a correlation coefficient of 0.66 (C). Template-based movie decoding was computed using the first viewing as the template and the second viewing as the training data. The clip was divided into subsections ranging from 2 to 30 clips. The confusion matrices for 4-way (D) and 8-way (E) decoding indicate strong decoding performance based on the higher diagonal elements of the matrix. Decoding accuracies were compared between VHD-DOT and MRI for all five splits of the movie clip, with VHD-DOT achieving a decoding performance above chance for each variation (F).

To test how repeatable these features are, we assessed the pairwise correlation between the first and second viewing of the movie clip (Fig. 6A). Here, we see higher t-values within the auditory and visual cortex, which is similarly reflected in the MRI results (Fig. 6B). The group-averaged single voxel (MNI coordinates: [42, 8, 22]) oxyhemoglobin time trace highlights the similarity between our VHD-DOT signal and the fMRI BOLD data with a correlation of 0.66 within the selected voxel (Fig. 6C). This correlation analysis indicates that we achieve repeatable VHD-DOT signals across multiple viewings of the same clip which is essential for future decoding studies. To further validate that VHD-DOT is suitable for decoding a stimulus from the measurements of brain function, we employed a template-matching decoding approach by subdividing the 10-minute movie clip into smaller clips. This allowed us to decode between 2, 4, 8, 15, and 30 unique movie clips. The aggregated confusion matrices for the 4-way (Fig. 6D) and 8-way (Fig. 6E) decoding paradigms show that decoding performance is well above chance, with an accuracy of 68.8% for 4-way decoding and 42.2% for 8-way decoding for VHD-DOT. Decoding performance for fMRI resulted in 75% accuracy for 4-way decoding and 65.6% for 8-way decoding. Performance in all iterations was well above chance, with VHD-DOT decoding performance on average 11.1 ± 7.4% lower than MRI decoding (Fig. 6F).

## 4 Discussion

This study presents a very high-density DOT system that covers most of the head with 255 sources and 252 detectors, resulting in nearly 10,000 measurements within a 4 cm source–detection distance. Spatial resolution improvements between the VHD-DOT and HD-DOT systems were quantified through point spread function simulations (Fig. 1). Raw data quality plots from the in vivo data indicate that we can measure the pulse signal, with SNR > 10 dB over the entire array of fibers, which is essential for our mapping of brain function (Fig. 2). Performance of VHD-DOT at mapping brain function, or encoding, was tested using a sequence of traditional functional localizers (Fig. 3) and naturalistic stimuli (Fig. 5). The feasibility of decoding neural responses from VHD-DOT data (Figs. 4and6) was also evaluated. Through both the encoding and decoding studies, we provided matching fMRI results for group-matched comparison. These results demonstrate that our VHD-DOT system has improved image quality over HD-DOT and can accurately and reliably map brain responses.

### 4.1 VHD-DOT design and validation

For DOT to be an impactful surrogate for fMRI, the image quality of DOT must closely resemble that of fMRI BOLD data. While HD-DOT has been shown to achieve comparable images with fMRI (Eggebrecht et al., 2014), further improvements to our image resolution and field of view are essential for mapping more complex stimuli. Previous HD-DOT systems typically used 13 mm between optode spacings (Eggebrecht et al., 2014) and achieved spatial resolutions between 13 and 16 mm depending on depth (Eggebrecht et al., 2012). The literature on these systems reported 1,200–3,000 source–detector measurements with posterior and lateral panels of the HD-DOT imaging cap. Here, we aimed to improve the spatial resolution of HD-DOT by reducing the between-optode spacing to less than 10 mm while simultaneously increasing our number of measurements to nearly 10,000. Through simulations with point targets, we show these improvements effectively decrease the full width at half maximum of our reconstructed points and improve image quality (Fig. 1B–E). Further, the localization error (Fig. 1G), effective resolution (Fig. 1H), and SNR (Fig. 1I) all demonstrate that an optode spacing of 9.75 mm would improve imaging quality, leading us to construct this VHD-DOT system. These data quality metrics use simulated noise from published HD-DOT data to account for physiological noise to avoid overstating the detection system’s fundamental signal to noise by only accounting for shot noise. However, a limitation is the exclusion of off diagonal elements in the covariance matrix which can be biologically relevant. While outside the scope of this paper, future development of simulation approaches can improve upon this limitation. Previous studies have shown in simulation and in vivo that further decreasing this optode spacing would result in additional improvements in image quality (Markow et al., 2025). However, the optode spacing of 6.5 mm presents additional optomechanical, optoelectrical, and ergonomic challenges when scaled to a whole-head imaging system with only marginal improvement to the image quality.

To balance our goal of whole-head imaging and an increased image resolution, we selected an optode spacing of 9.75 mm with 252 sources and 255 detectors distributed in a grid pattern across the head (Fig. 2A, B). This doubled the number of sources and detectors of previous HD-DOT systems (Eggebrecht et al., 2014;Tripathy et al., 2024), resulting in a higher measurement density and a larger field of view (Fig. 1J). The VHD-DOT system’s field of view covers occipital and temporal regions as well as the parietal and frontal areas. This expanded coverage is critical for comprehensive brain mapping and of particular value for researching the semantic system, which encompasses large areas of the brain. Spring-loaded fiber tips and a lacing system with a ratcheting front mechanism were designed to allow for this extensive coverage while accounting for differences in head shapes and emphasizing participant comfort. The spring-loaded fiber tips improved the optode-to-scalp coupling by conforming to the head better than our previous rubber-loaded optodes. Our lacing/ratchet mechanism distributed pressure more evenly across the head than the previous hook-and-loop fastener system (Eggebrecht et al., 2014;Tripathy et al., 2024). While quantitative metrics for rating participant comfort were not employed in this study, participants were able to complete lengthy scan sessions, which consisted of 1–1.5 hours in the cap, including the cap fit procedure. Future work can assess these system changes’ impact on participant comfort through surveys during the scanning sessions. While some aspects of the system are reasonably difficult to disseminate (the electo-optic console), other aspects directly related to the very high-density imaging arrays are straightforward to disseminate. For this, we are sharing the fiber part numbers, and all of the components of the cap. In detail, we have included the Standard Triangle Language (STL) files for 3D printing the cap, spring-loaded fiber tips, and ratchet housing as available for download (see Data and Code Availability statement). These files can facilitate the widespread use of spring-loaded fiber tips, ratchet-based lacing mechanisms, and 3D printing approaches to constructing fiber-based DOT systems. The adoption of these cap improvements can support further advancements in the field.

The optode-to-scalp coupling can be evaluated from the raw data quality plots, including the mean band-limited pulse SNR plot (Fig. 2G). Here, the SNR appears even across the cap with a maximum value of approximately 30 dB. Areas with lower SNR include the motor pad’s posterior region and the cap’s sides, where the visual panel connects to the side panels. These three regions tend to display lower pulse SNR values due to worse optode-to-scalp coupling from the cap’s curvature, which mismatches the curvature of the scalp in some participants. However, there is still sufficient SNR for imaging as our other raw data quality plots, including the Fourier spectra (Fig. 2D) and time traces (Fig. 2E), suggest that we can measure changes in hemoglobin based on visualizing the participant’s pulse. The effectiveness of the VHD-DOT system was also evaluated through the light fall-off to visualize the light levels as a function of source–detector separation. First through fifth nearest neighbor measurement pairs were retained with measurements above the noise floor. From the light falloff, we see the two-pass encoding pattern extended the system’s dynamic range to ~10^6^, similar to previous DOT systems (Markow, Trobaugh, et al., 2023;Tripathy et al., 2024). Measurement pairs were further evaluated using the histogram (Fig 2F) to visualize the proportion of measurements retained after the temporal standard deviation threshold of 7.5% was applied. This proportion of retained measurements was used for participant selection, as participants with less than 80% of retained measurements were excluded from the study. As only two participants were excluded based on data quality, we can infer that the VHD-DOT system is effective across a range of adult participants. While this cap is unsuitable for children due to the adult-sized cap, further iterations of VHD-DOT systems could be designed for pediatric imaging by using a smaller target head size and shape that would likely use a smaller number of sources and detectors but maintain the array density to keep the improvements in image quality presented herein. Overall, this system can acquire many source–detector measurements with data quality sufficient for measuring changes in hemoglobin.

Collecting robust, repeatable signals is essential for functional neuroimaging studies. A battery of functional localizers was selected to assess the image quality of the VHD-DOT system in comparison with the gold standard of fMRI. The t-statistic group-level and single-subject beta maps generated from these localizer tasks validate the VHD-DOT system image quality. Word hearing and verb generation tasks elicited auditory and language activations similar to fMRI (Fig. 3A, B). Retinotopic and motor mapping activate the left and right visual and motor cortex (Fig. 3C, D). The t-statistic values for the VHD-DOT data are comparable with fMRI, suggesting that VHD-DOT can be used as a high-fidelity surrogate for fMRI. The overlap maps generated from the binarized group-average data (Supplementary Fig. S9) continue to analyze the alignment between the VHD-DOT and fMRI data. The word hearing and retinotopic maps highlight the strong comparison between the modalities, which is further validated by the Dice coefficient values of 0.54 (word hearing), 0.23 (right visual), and 0.40 (left visual) when the group-average maps used a threshold of 25% of the maximum T-statistic value (Supplementary Table S2). The dispersed activations of the verb generation task led to poorer performance in the direct comparison between modalities. However, the Dice coefficient of 0.30 when using a threshold of 25% of the maximum T-statistic still yields an impressive comparison between the VHD-DOT and fMRI systems. Specifically for the verb generation tasks, there appears to be a shift in the activations toward the front for VHD-DOT relative to fMRI. One challenge with these comparisons is that as the resolution improves, the need for precision co-registration also needs to improve. Finally, the motor task exhibited the lowest overlap between the modalities, which is congruent with the fairly consistently lower signal to noise we find in the dorsal panel of the cap (Fig. 2G). Future developments to the VHD-DOT imaging systems should focus on optimizing the dorsal panel to improve the optode-to-scalp coupling. This will likely involve re-thinking the way the dorsal panel attaches to the side and back panels of the cap. As noted in the results analyzing the overlap and Dice analysis between the VHD-DOT and fMRI data (Supplementary Fig. S9), as the resolution of VHD-DOT improves, the errors in co-registration to anatomy become highlighted. This can be seen most clearly for the verb generation task, where the overlap is significantly higher for maps with 25% of the maximum value compared with the 50% maximum value maps. In those particular maps, there appears to be an anterior-posterior shift in the VHD-DOT data when compared with fMRI. The DOT field in general will benefit from improved, more precise co-registration of imaging arrays to subject anatomy (Ferradal et al., 2014). As better co-registration tools become available, we anticipate the congruence between VHD-DOT and fMRI to improve.

Block average time traces from localizer-defined regions of interest further reinforce the quality of the VHD-DOT signals for oxyhemoglobin, deoxyhemoglobin, and total hemoglobin. Overall, the functional localizer validation tasks highlight the extended coverage of the VHD-DOT imaging system throughout superficial cortex. While all optical neuroimaging techniques suffer from depth limitations that impact the field of view, this is far more detrimental to sparse imaging systems. As shown through simulations, the VHD-DOT system presented here offers improved image quality across multiple depths compared with HD-DOT (Fig. 1F–I). This highlights the higher spatial resolution for VHD-DOT over HD-DOT and traditional sparse fNIRS imaging systems. However, while the VHD-DOT system cannot yet equal the high spatial resolution of fMRI, our system validation proves that VHD-DOT can improve resolution which improves its performance as a surrogate for fMRI in functional neuroimaging tasks.

### 4.2 Mapping of naturalistic stimuli

To map more complex, naturalistic stimuli using the VHD-DOT system, we started with a previously validated movie-viewing task (Fishell et al., 2019;Tripathy et al., 2024). Movie viewing offers a more engaging, ecologically valid functional imaging task akin to what participants would do in their everyday life, instead of a controlled laboratory setting. This provides more insight into the brain in a natural state while still evoking a repeatable neural response (Andric et al., 2016;Hasson et al., 2010;Hasson et al., 2004). Movie viewing additionally allows for the parallel mapping of multiple sensory pathways through feature regressor analysis (Bartels & Zeki, 2004). The movie selection is vital for this type of analysis, as features must be unique and repeated throughout the movie (Tripathy et al., 2024). Here, we used a previously validated 10-minute clip from The Good, The Bad, and The Ugly (Fishell et al., 2019). The participants viewed the clip a total of four times across the VHD-DOT and MRI data collection. To minimize the habituation effects of the repeated task on the hemodynamic responses in the brain, we kept to a minimum of 3 weeks between imaging sessions. While habituation effects are important to consider, we have seen no effects of day-to-day habituation to reductive tasks like visual stimuli in our other studies (Fishell et al., 2019;Markow et al., 2025;Tripathy et al., 2021,^2024^,^2025^). The effects of repeated tasks on hemodynamic responses could be assessed in future studies.

Five features, including audio envelope, luminance, speech, faces, and hands, were extracted from the movie clip to be correlated with the VHD-DOT and fMRI signals. The group-level feature regressor maps for speech and audio envelope display higher correlation values within the temporal cortex. The low-level luminance regressor, designed to evoke visual activations, correlated with voxels within the occipital lobe. The hands feature regressor similarly evokes visual activations within the occipital lobe and the middle temporal visual area associated with motion (Born & Bradley, 2005). The face regressor correlated primarily to voxels within the superior temporal sulcus, a region associated with face processing (Tsao & Livingstone, 2008). The activated regions from the speech regressor appear similar to those activated during our word-hearing localizer task, indicating that a movie-viewing task can map similar tasks and areas as those accessed with block-design tasks while having the advantage of being more engaging and ecologically valid. Outside the laboratory, we rarely encounter sensory information in isolation but experience multiple sensations simultaneously, such as sound and visuals, when watching a movie. Overall, this highlights that the VHD-DOT system is suitable for naturalistic imaging paradigms with high signal fidelity and a field of view that covers large areas in the cortex.

The similarities between the voxel responses and the feature regressors are further validated by plotting group-averaged single-voxel VHD-DOT and BOLD signals along with the speech and hands regressors. The correlation value between the signals and the regressors is 0.47 for speech and 0.35 for hands, comparable with the MRI correlation values of 0.20 for speech and 0.37 for hands. While these correlation values are less than 0.5, they are still considered high correlation values in the context of naturalistic imaging (Tripathy et al., 2024). As previously shown, low inter-run correlation values can still lead to high statistical performance and strong test–retest reliability (Hasson et al., 2004,^2010^). Additionally, these low correlation values can still support excellent encoding and decoding performance when considering all cortical voxels (LeBel et al., 2023). Previous HD-DOT movie-viewing studies using audiovisual (Fishell et al., 2019;Tripathy et al., 2024) and visual-only (Markow, Tripathy, et al., 2023) stimuli have lacked direct validation using subject-matched fMRI data. Here, we further highlight the similarity between our VHD-DOT signals and fMRI to validate the imaging system as a surrogate for fMRI, focusing on naturalistic imaging tasks.

### 4.3 Decoding of stimuli with VHD-DOT data

Recent functional neuroimaging studies take naturalistic tasks a step further from brain mapping, or encoding, to decoding, often predicting what the participant was seeing (Huth, Lee, et al., 2016;Markow, Tripathy, et al., 2023;Nishimoto et al., 2011;Tripathy et al., 2021) or hearing (LeBel et al., 2023;Tang et al., 2023) during the task. Complex, naturalistic decoding paradigms are frequently accomplished using fMRI BOLD signals. Still, the lack of portability of MRI makes extrapolating these techniques to brain–computer interfaces or clinical applications a challenge. The potential portability and wearability of fNIRS and DOT make them possible fMRI surrogates for these decoding studies. However, the low spatial resolution of fNIRS limits the ability to decode naturalistic tasks, often leading to decoding studies that coarsely classify tasks between 2 and 6 categories (Emberson et al., 2017;Hong & Santosa, 2016;Yoo et al., 2021). Although work is being done with semantic decoding using fNIRS signals (Zinszer et al., 2018), poor image quality remains a challenge. Commonly, fNIRS studies are completed using systems with <100 channels, compared with the nearly 10,000 channels of the VHD-DOT system. The improvements in spatial resolution for HD-DOT have allowed for naturalistic visual-only decoding using silent movie clips (Markow, Tripathy, et al., 2023). Here, we established the feasibility of decoding using VHD-DOT by highlighting the collection of repeatable and discriminable signals in localizer and naturalistic imaging tasks.

A template-matching approach for stimulus identification was selected to decode our functional localizer responses. Six templates constructed from three blocks of each task were used for trial-based classification with less than 20 seconds of data. With a decoding accuracy of 76.4% across all participants, this approach highlights the VHD-DOT system’s ability to collect highly repeatable and discernible signals. This accuracy is well above chance (16.7%) and comparable with the decoding accuracy of 87.5% from fMRI data in the same set of participants. From the group summed confusion matrices, we see that most inaccuracies are from the visual and motor tasks. This is likely due to misclassifying these tasks’ left and right conditions, as the activations can be spatially similar due to noise. Alternatively, the word hearing and verb generation tasks evoke spatially dissimilar activations and, as such, were decoded with near-perfect accuracy across all participants. Decoding performance was further assessed individually for each subject, as data quality and participant compliance can significantly impact results. Three participants achieved the same decoding accuracy for both VHD-DOT and fMRI. This result further supports the argument that VHD-DOT can be used as a surrogate for fMRI. Three of the remaining participants had a less than 15% difference, while two had a greater than 20% difference between VHD-DOT and fMRI. Poor VHD-DOT signal quality from low optode-to-scalp coupling or high motion may have contributed to this decline in decoding performance. However, this template-matching experiment further validates the VHD-DOT system by emphasizing the collection of robust, discriminable signals from our functional localizer data collection.

The decoding of naturalistic stimuli takes our decoding approach a step further by classifying simultaneous auditory and visual information. To first establish the repeatability of the movie-viewing data, the pairwise correlation between the two viewings of the clip was computed. The higher correlation regions within the visual and auditory cortex are consistent with past findings in both DOT (Fishell et al., 2019;Tripathy et al., 2024) and MRI (Hasson et al., 2010;Hasson et al., 2004), validating that our system can achieve repeatable signals using a naturalistic stimulus. The signals from VHD-DOT and fMRI were also remarkably similar, with a correlation of 0.66 for a single group-averaged voxel. A template-based decoding approach was used to validate the repeatability of the VHD-DOT signal. Here, the 10-minute movie was divided into 2, 4, 8, 15, and 30 unique movie clips where the first viewing was considered the template, and the second viewing was used as the test data. Decoding performance was consistently well above chance and comparable with fMRI. For the 4-way and 8-way decoding tasks, VHD-DOT performed with an accuracy of 68.8% and 42.2%, respectively, compared with the 75% and 65.6% decoding accuracy for fMRI. Across all audiovisual movie decoding tasks (2-, 4-, 8-, 15-, and 30-way decoding), the VHD-DOT decoding performance was 11.1 ± 7.4% lower than the MRI decoding performance. This difference is driven by the image resolution and field of view of fMRI compared with VHD-DOT. The greatest discrepancy between fMRI and VHD-DOT occurred with the eight-way decoding. This could be attributed to VHD-DOT requiring more time points for accurate decoding compared with fMRI as the 8-way decoding uses only 55 seconds of data. However, the decoding performance for VHD-DOT remained well above chance in all decoding cases, implying that the VHD-DOT system is capable of accurately decoding naturalistic stimuli using a template-based approach. This approach builds upon the template decoding presented inMarkow, Tripathy, et al. (2023)for visual decoding and extends it to audiovisual decoding using VHD-DOT. This work establishes the feasibility of naturalistic decoding using VHD-DOT for future experiments with model-based decoding approaches.

### 4.4 Future directions

The development and validation of our VHD-DOT imaging system represent an advance in optical neuroimaging, more closely matching the gold standard of fMRI than previous work. The improved image quality and expanded field of view offer a high-resolution, whole-head imaging system capable of imaging studies that are more naturalistic than the scanning environment of MRI. Numerous future studies are now viable with the VHD-DOT imaging system, including studies of semantic systems that rely on high-quality whole-head imaging. While previous fNIRS studies have sought to evaluate and decode semantic representations (Rybář et al., 2021;Zinszer et al., 2018), the advancements from VHD-DOT will increase the channel count by 100-fold and allow more complex stimuli and analysis pipelines from the fMRI research community (Nishimoto et al., 2011;Tang et al., 2023). In particular,Tang et al. (2023)investigate the impact on semantic auditory decoding when smoothing the fMRI data to match that of HD-DOT. This preliminary result illustrates that decoding performance would decline but remain sufficient, providing promise for semantic decoding studies using VHD-DOT. This further demonstrates the neuroimaging community’s interest in wearable optical imaging methods. VHD-DOT can be used for more naturalistic imaging studies, such as expanding upon previous fNIRS work using video games (Matsuda & Hiraki, 2006; Tachtsidis & Papaioannou, 2013), face-to-face interactions (Hirsch et al., 2017,^2020^), or skilled activities (Vanzella et al., 2019). Although the VHD-DOT system in this paper uses a relatively fixed position with the fibers coming down from a structure above the subject, previous fiber-based HD-DOT systems have been adapted to more flexible positioning, including portable clinical systems that can be taken to the bedside or taken into low-resource settings (Ferradal et al., 2016;Fishell et al., 2020;Perdue et al., 2019). VHD arrays could be implemented in these settings. Compared with fMRI, the VHD-DOT in this paper allows the participant to be positioned in a chair in a sitting position with an open-air scanning environment, with the absence of noise. This allows for the use of arm and leg movements, including motor imitation tasks, and direct person–person interactions with natural visual views and natural three-dimensional acoustics. These tasks are either challenging, or impossible, to complete using fMRI.

Additional VHD-DOT data collection and processing optimization are ongoing, including improved co-registration between imaging sessions for multi-day scanning to allow for complex, precision-focused experiments (Bajracharya et al., 2023). Precision data collection techniques, coupled with subject-specific head modeling, allow for the precise placement of optodes onto the scalp during both data collection and analysis. During data collection, an efficient cap fit procedure emphasizes consistent cap placement, subject comfort, and data quality. While our method is efficient, often requiring less than 15 minutes, future work includes developing a patient and pediatric-friendly cap fit procedure to ensure optical placement. To guide this development, we can follow previously defined pediatric procedures used for our HD-DOT imaging system (Tripathy et al., 2024). Future directions for locating cap placement include using advanced photogrammetry approaches to record the optode positions (Mazzonetto et al., 2022). When anatomical and functional MRI data are unavailable, the subject-specific head modeling techniques outlined here can still be applied using an MRI atlas-derived head model (Ferradal et al., 2014). Additionally, the functional alignment using fMRI and DOT data can be performed using group-averaged fMRI data as a target for the DOT-reconstructed data (Tripathy et al., 2024). This combination of anatomical and functional alignment provides increased localization for our optode positioning and helps drive our registration between the fMRI and DOT data. Future work could include a more rigorous assessment of the subject-specific head modeling steps to evaluate the improvement, but this is outside the scope of the current study. While subject-specific MRI data allow for the most accurate approach to head modeling, these methods are still applicable when MRI data are unavailable. Future work with the VHD-DOT imaging system will build upon this subject-specific head modeling technique to improve co-registration between imaging sessions and establish optimal techniques for head modeling in the absence of MRI data.

For VHD-DOT to be used in a truly naturalistic environment, future work must be dedicated to extending VHD arrays into wearable systems. This would allow for greater movement, and increasingly naturalistic and flexible paradigms during imaging sessions. Wearable fNIRS and DOT systems have been on the rise and have been successful in imaging adults (Chitnis et al., 2016;Uchitel et al., 2022;Vidal-Rosas et al., 2020) and children (Bulgarelli et al., 2023;Frijia et al., 2021). These wearable systems are largely built on the foundation of the literature from fiber-based imaging systems (Vidal-Rosas et al., 2023). While not all fiber-based systems were distributed beyond the groups that developed them (Eggebrecht et al., 2014;White & Culver, 2010;Zeff et al., 2007), several systems have been (Franceschini et al., 2006;Schmitz et al., 2000). However, the trend for commercialization is clearly focused on wearable HD-DOT systems (Collins-Jones et al., 2024;O’Brien et al., 2024;Piper et al., 2014;Vidal-Rosas et al., 2023). Future system optimization for VHD-DOT will be best pursued with wearable systems better positioned to continue pushing the boundaries of naturalistic imaging paradigms. These wearable systems are based on the literature from fiber-based imaging systems, which often are not distributed beyond the groups that developed them. Therefore, future system optimization can focus on developing a wearable VHD-DOT system for improved image quality, portability, and dissemination to other research groups. By transitioning these advancements into a wearable system, the imaging system can be better deployed across the neuroimaging community to continue pushing the boundaries of naturalistic imaging paradigms. Overall, our work developing and validating the VHD-DOT imaging system highlights its improved image resolution and whole-head imaging capabilities compared with fMRI. This system is an important foundation for future naturalistic neuroimaging studies, including semantic brain mapping and decoding for studying neuroscience, which has potential clinical applications.

## Supplementary Material

Supplementary Material

## Data Availability

To enable further analysis by other groups, our VHD-DOT data and STL 3D printing files for cap development are available publicly through OXI and distributed through Washington University in St. Louis. The MRI data are available upon reasonable request by contacting the corresponding author. The code for processing these data is publicly available through NITRC (https://www.nitrc.org/projects/neurodot/). Additional code specific to these data can be obtained via GitHub (https://github.com/WUSTL-CulverLab/VHD-System-Paper).

## References

[IMAG.a.54-b1] Andric , M. , Goldin-Meadow , S. , Small , S. L. , & Hasson , U. ( 2016 ). Repeated movie viewings produce similar local activity patterns but different network configurations . NeuroImage , 142 , 613 – 627 . 10.1016/j.neuroimage.2016.07.061 27492251

[IMAG.a.54-b2] Bajracharya , A. , Wilhelm , D. , Markow , Z. , Fogarty , M. , Fehner , W. , Peelle , J. E. , Hershey , T. , & Culver , J. P. ( 2023 ). Precision functional mapping of cortical activity using high-density diffuse optical tomography (HD-DOT) . In Biophotonics Congress: Optics in the Life Sciences 2023 (OMA, NTM, BODA, OMP, BRAIN), Vancouver, British Columbia (Technical Digest Series). Optica Publishing Group . 10.1364/boda.2023.jtu4b.15

[IMAG.a.54-b3] Barbour , R. L. , Graber , H. L. , Aronson , R. , & Lubowky , J. ( 1991 ). Imaging of subsurface regions of random media by remote sensing. In Proc. SPIE 1431, Time-Resolved Spectroscopy and Imaging of Tissues, 1 May . SPIE . 10.1117/12.44190

[IMAG.a.54-b4] Bartels , A. , & Zeki , S. ( 2004 ). Functional brain mapping during free viewing of natural scenes . Human Brain Mapping , 21 ( 2 ), 75 – 85 . 10.1002/hbm.10153 14755595 PMC6872023

[IMAG.a.54-b5] Boas , D. A. , & Yodh , A. G. ( 1997 ). Spatially varying dynamical properties of turbid media probed with diffusing temporal light correlation . Journal of the Optical Society of America A , 14 ( 1 ), 192 – 215 . 10.1364/JOSAA.14.000192

[IMAG.a.54-b6] Born , R. T. , & Bradley , D. C. ( 2005 ). Structure and function of visual area MT . Annual Review of Neuroscience , 28 , 157 – 189 . 10.1146/annurev.neuro.26.041002.131052 16022593

[IMAG.a.54-b7] Brainard , D. H. ( 1997 ). The psychophysics toolbox . Spatial Vision , 10 , 433 – 436 . 10.1163/156856897x00357 9176952

[IMAG.a.54-b8] Bulgarelli , C. , Pinti , P. , Aburumman , N. , & Jones , E. J. H. ( 2023 ). Combining wearable fNIRS and immersive virtual reality to study preschoolers’ social development: A proof-of-principle study on preschoolers’ social preference . Oxford Open Neuroscience , 2 , kvad012 . 10.1093/oons/kvad012 38596237 PMC10913823

[IMAG.a.54-b9] Chang , W. T. , Jääskeläinen , I. P. , Belliveau , J. W. , Huang , S. , Hung , A. Y. , Rossi , S. , & Ahveninen , J. ( 2015 ). Combined MEG and EEG show reliable patterns of electromagnetic brain activity during natural viewing . NeuroImage , 114 , 49 – 56 . 10.1016/j.neuroimage.2015.03.066 25842290 PMC4446182

[IMAG.a.54-b10] Chitnis , D. , Cooper , R. J. , Dempsey , L. , Powell , S. , Quaggia , S. , Highton , D. , Elwell , C. , Hebden , J. C. , & Everdell , N. L. ( 2016 ). Functional imaging of the human brain using a modular, fibre-less, high-density diffuse optical tomography system . Biomedical Optics Express , 7 , 4275 . 10.1364/boe.7.004275 27867731 PMC5102535

[IMAG.a.54-b11] Collins-Jones , L. H. , Gossé , L. K. , Blanco , B. , Bulgarelli , C. , Siddiqui , M. , Vidal-Rosas , E. E. , Duobaitė , N. , Nixon-Hill , R. W. , Smith , G. , Skipper , J. , Sargent , T. , Powell , S. , Everdell , N. L. , Jones , E. J. H. , & Cooper , R. J. ( 2024 ). Whole-head high-density diffuse optical tomography to map infant audio-visual responses to social and non-social stimuli . Imaging Neuroscience , 2 , 1 – 19 . 10.1162/imag_a_00244 PMC12272203 40800512

[IMAG.a.54-b12] Dehghani , H. , Eames , M. E. , Yalavarthy , P. K. , Davis , S. C. , Srinivasan , S. , Carpenter , C. M. , Pogue , B. W. , & Paulsen , K. D. ( 2008 ). Near infrared optical tomography using NIRFAST: Algorithm for numerical model and image reconstruction . Communications in Numerical Methods in Engineering , 25 ( 6 ), 711 – 732 . 10.1002/cnm.1162 20182646 PMC2826796

[IMAG.a.54-b13] Deniz , F. , Nunez-Elizalde , A. O. , Huth , A. G. , & Gallant , J. L. ( 2019 ). The representation of semantic information across human cerebral cortex during listening versus reading is invariant to stimulus modality . The Journal of Neuroscience , 39 , 7722 – 7736 . 10.1523/jneurosci.0675-19.2019 31427396 PMC6764208

[IMAG.a.54-b14] Desai , M. , Holder , J. , Villarreal , C. , Clark , N. , Hoang , B. , & Hamilton , L. S. ( 2021 ). Generalizable EEG encoding models with naturalistic audiovisual stimuli . The Journal of Neuroscience , 41 ( 43 ), 8946 – 8962 . 10.1523/JNEUROSCI.2891-20.2021 34503996 PMC8549533

[IMAG.a.54-b15] Durduran , T. , Choe , R. , Baker , W. B. , & Yodh , A. G. ( 2010 ). Diffuse optics for tissue monitoring and tomography . Reports on Progress in Physics , 73 ( 7 ), 076701 . 10.1088/0034-4885/73/7/076701 26120204 PMC4482362

[IMAG.a.54-b16] Eggebrecht , A. T. , Ferradal , S. L. , Robichaux-Viehoever , A. , Hassanpour , M. S. , Dehghani , H. , Snyder , A. Z. , Hershey , T. , & Culver , J. P. ( 2014 ). Mapping distributed brain function and networks with diffuse optical tomography . Nature Photonics , 8 ( 6 ), 448 – 454 . 10.1038/nphoton.2014.107 25083161 PMC4114252

[IMAG.a.54-b17] Eggebrecht , A. T. , White , B. R. , Ferradal , S. L. , Chen , C. , Zhan , Y. , Snyder , A. Z. , Dehghani , H. , & Culver , J. P. ( 2012 ). A quantitative spatial comparison of high-density diffuse optical tomography and fMRI cortical mapping . NeuroImage , 61 ( 4 ), 1120 – 1128 . 10.1016/j.neuroimage.2012.01.124 22330315 PMC3581336

[IMAG.a.54-b18] Emberson , L. L. , Zinszer , B. D. , Raizada , R. D. S. , & Aslin , R. N. ( 2017 ). Decoding the infant mind: Multivariate pattern analysis (MVPA) using fNIRS . PLoS One , 12 , 0172500 . 10.1371/journal.pone.0172500 PMC5398514 28426802

[IMAG.a.54-b19] Esteban , O. , Blair , R. , Markiewicz , C. J. , Berleant , S. L. , Moodie , C. , Ma , F. , Isik , A. I. , Erramuzpe , A. , Kent , J. D. , Goncalves , M. , DuPre , E. , Sitek , K. R. , Gomez , D. E. P. , Lurie , D. J. , Ye , Z. , Poldrack , R. A. , & Gorgolewski , K. J. ( 2018 ). fMRIPrep 22.0.2. Software . Zenodo. 10.5281/zenodo.852659

[IMAG.a.54-b20] Esteban , O. , Markiewicz , C. , Blair , R. W. , Moodie , C. , Isik , A. I. , Erramuzpe Aliaga , A. , Kent , J. , Goncalves , M. , DuPre , E. , Snyder , M. , Oya , H. , Ghosh , S. , Wright , J. , Durnez , J. , Poldrack , R. , & Gorgolewski , K. J. ( 2018 ). fMRIPrep: A robust preprocessing pipeline for functional MRI . Nature Methods , 16 ( 1 ), 111 – 116 . 10.1038/s41592-018-0235-4 30532080 PMC6319393

[IMAG.a.54-b21] Ferradal , S. L. , Eggebrecht , A. T. , Hassanpour , M. , Snyder , A. Z. , & Culver , J. P. ( 2014 ). Atlas-based head modeling and spatial normalization for high-density diffuse optical tomography: In vivo validation against fMRI . NeuroImage , 85 , 117 – 126 . 10.1016/j.neuroimage.2013.03.069 23578579 PMC4433751

[IMAG.a.54-b22] Ferradal , S. L. , Liao , S. M. , Eggebrecht , A. T. , Shimony , J. S. , Inder , T. E. , Culver , J. P. , & Smyser , C. D. ( 2016 ). Functional imaging of the developing brain at the bedside using diffuse optical tomography . Cerebral Cortex , 26 , 1558 – 1568 . 10.1093/cercor/bhu320 25595183 PMC4785947

[IMAG.a.54-b23] Finn , E. S. , & Bandettini , P. A. ( 2021 ). Movie-watching outperforms rest for functional connectivity-based prediction of behavior . NeuroImage , 235 , 117963 . 10.1016/j.neuroimage.2021.117963 33813007 PMC8204673

[IMAG.a.54-b24] Fischl , B. ( 2012 ). FreeSurfer . NeuroImage , 62 ( 2 ), 774 – 781 . 10.1016/j.neuroimage.2012.01.021 22248573 PMC3685476

[IMAG.a.54-b25] Fishell , A. K. , Arbeláez , A. M. , Valdés , C. P. , Burns-Yocum , T. M. , Sherafati , A. , Richter , E. J. , Torres , M. , Eggebrecht , A. T. , Smyser , C. D. , & Culver , J. P. ( 2020 ). Portable, field-based neuroimaging using high-density diffuse optical tomography . NeuroImage , 215 , 116541 . 10.1016/j.neuroimage.2020.116541 31987995

[IMAG.a.54-b26] Fishell , A. K. , Burns-Yocum , T. M. , Bergonzi , K. M. , Eggebrecht , A. T. , & Culver , J. P. ( 2019 ). Mapping brain function during naturalistic viewing using high-density diffuse optical tomography . Scientific Reports , 9 ( 1 ), 11115 . 10.1038/s41598-019-45555-8 31366956 PMC6668456

[IMAG.a.54-b27] Franceschini , M. A. , Joseph , D. , Huppert , T. , Diamond , S. , & Boas , D. ( 2006 ). Diffuse optical imaging of the whole head . Journal of Biomedical Optics , 11 ( 5 ), 054007 . 10.1117/1.2363365 17092156 PMC2637816

[IMAG.a.54-b28] Frijia , E. M. , Billing , A. , Lloyd-Fox , S. , Vidal Rosas , E. , Collins-Jones , L. , Crespo-Llado , M. M. , Amadó , M. P. , Austin , T. , Edwards , A. , Dunne , L. , Smith , G. , Nixon-Hill , R. , Powell , S. , Everdell , N. L. , & Cooper , R. J. ( 2021 ). Functional imaging of the developing brain with wearable high-density diffuse optical tomography: A new benchmark for infant neuroimaging outside the scanner environment . NeuroImage , 225 , 117490 . 10.1016/j.neuroimage.2020.117490 33157266

[IMAG.a.54-b29] Gal , S. , Coldham , Y. , Tik , N. , Bernstein-Eliav , M. , & Tavor , I. ( 2022 ). Act natural: Functional connectivity from naturalistic stimuli fMRI outperforms resting-state in predicting brain activity . NeuroImage , 258 , 119359 . 10.1016/j.neuroimage.2022.119359 35680054

[IMAG.a.54-b30] Gordon , E. M. , Laumann , T. O. , Gilmore , A. W. , Newbold , D. J. , Greene , D. J. , Berg , J. J. , Ortega , M. , Hoyt-Drazen , C. , Gratton , C. , Sun , H. , Hampton , J. M. , Coalson , R. S. , Nguyen , A. L. , McDermott , K. B. , Shimony , J. S. , Snyder , A. Z. , Schlaggar , B. L. , Petersen , S. E. , Nelson , S. M. , & Dosenbach , N. U. F. ( 2017 ). Precision functional mapping of individual human brains . Neuron , 95 ( 4 ), 791.7 – 807.e7 . 10.1016/j.neuron.2017.07.011 28757305 PMC5576360

[IMAG.a.54-b31] Gorgolewski , K. , Burns , C. D. , Madison , C. , Clark , D. , Halchenko , Y. O. , Waskom , M. L. , & Ghosh , S. ( 2011 ). Nipype: A flexible, lightweight and extensible neuroimaging data processing framework in Python . Frontiers in Neuroinformatics , 5 , 13 . 10.3389/fninf.2011.00013 21897815 PMC3159964

[IMAG.a.54-b32] Gorgolewski , K. J. , Esteban , O. , Markiewicz , C. J. , Ziegler , E. , Ellis , D. G. , Notter , M. P. , Jarecka , D. , Johnson , H. , Burns , C. , Manhães-Savio , A. , Hamalainen , C. , Yvernault , B. , Salo , T. , Jordan , K. , Goncalves , M. , Waskom , M. , Clark , D. , Wong , J. , Loney , F. ,… Ghosh , S . ( 2018 ). Nipype Software . Zenodo. 10.5281/zenodo.596855

[IMAG.a.54-b33] Gregg , N. M. , White , B. R. , Zeff , B. W. , Berger , A. J. , & Culver , J. P. ( 2010 ). Brain specificity of diffuse optical imaging: Improvements from superficial signal regression and tomography . Frontiers in Neuroenergetics , 2 , 14 . 10.3389/fnene.2010.00014 20725524 PMC2914577

[IMAG.a.54-b34] Hanli , L. , Boas , D. A. , Yutao , Z. , Yodh , A. G. , & Chance , B. ( 1995 ). Determination of optical properties and blood oxygenation in tissue using continuous NIR light . Physics in Medicine & Biology , 40 ( 11 ), 1983 . 10.1088/0031-9155/40/11/015 8587945

[IMAG.a.54-b35] Hassanpour , M. S. , White , B. R. , Eggebrecht , A. T. , Ferradal , S. L. , Snyder , A. Z. , & Culver , J. P. ( 2014 ). Statistical analysis of high density diffuse optical tomography . NeuroImage , 85 , 104 – 116 . 10.1016/j.neuroimage.2013.05.105 23732886 PMC4097403

[IMAG.a.54-b36] Hasson , U. , Malach , R. , & Heeger , D. J. ( 2010 ). Reliability of cortical activity during natural stimulation . Trends in Cognitive Sciences , 14 , 40 – 48 . 10.1016/j.tics.2009.10.011 20004608 PMC2818432

[IMAG.a.54-b37] Hasson , U. , Nir , Y. , Levy , I. , Fuhrmann , G. , & Malach , R. ( 2004 ). Intersubject synchronization of cortical activity during natural vision . https://www.science.org 10.1126/science.1089506 15016991

[IMAG.a.54-b38] Haufe , S. , DeGuzman , P. , Henin , S. , Arcaro , M. , Honey , C. J. , Hasson , U. , & Parra , L. C. ( 2018 ). Elucidating relations between fMRI, ECoG, and EEG through a common natural stimulus . NeuroImage , *179* , 79–91. 10.1016/j.neuroimage.2018.06.016 PMC6063527 29902585

[IMAG.a.54-b86] Hirsch , J. , Tiede , M. , Zhang , X. , Noah , J. A. , Salama-Manteau , A. , & Biriotti , M. ( 2020 ). Interpersonal agreement and disagreement during face-to-face dialogue: An fNIRS investigation . Frontiers in Human Neuroscience , 14 , 606397. 10.3389/fnhum.2020.606397 PMC7874076 33584223

[IMAG.a.54-b39] Hirsch , J. , Zhang , X. , Noah , J. A. , & Ono , Y. ( 2017 ). Frontal temporal and parietal systems synchronize within and across brains during live eye-to-eye contact . NeuroImage , 157 , 314 – 330 . 10.1016/j.neuroimage.2017.06.018 28619652 PMC5863547

[IMAG.a.54-b40] Hong , K. S. , & Santosa , H. ( 2016 ). Decoding four different sound-categories in the auditory cortex using functional near-infrared spectroscopy . Hearing Research , 333 , 157 – 166 . 10.1016/j.heares.2016.01.009 26828741

[IMAG.a.54-b41] Huth , A. G. , De Heer , W. A. , Griffiths , T. L. , Theunissen , F. E. , & Gallant , J. L. ( 2016 ). Natural speech reveals the semantic maps that tile human cerebral cortex . Nature , 532 ( 7600 ), 453 – 458 . 10.1038/nature17637 27121839 PMC4852309

[IMAG.a.54-b42] Huth , A. G. , Lee , T. , Nishimoto , S. , Bilenko , N. Y. , Vu , A. T. , & Gallant , J. L. ( 2016 ). Decoding the semantic content of natural movies from human brain activity . Frontiers in Systems Neuroscience , 10 , 81 . 10.3389/fnsys.2016.00081 27781035 PMC5057448

[IMAG.a.54-b43] Huth , A. G. , Nishimoto , S. , Vu , A. T. , & Gallant , J. L. ( 2012 ). A continuous semantic space describes the representation of thousands of object and action categories across the human brain . Neuron , 76 ( 6 ), 1210 – 1224 . 10.1016/j.neuron.2012.10.014 23259955 PMC3556488

[IMAG.a.54-b44] Jain , S. , & Huth , A. G. ( 2018 ). Incorporating context into language encoding models for fMRI . bioRxiv . 10.1101/327601

[IMAG.a.54-b45] Jermyn , M. , Ghadyani , H. , Mastanduno , M. A. , Turner , W. , Davis , S. C. , Dehghani , H. , & Pogue , B. W. ( 2013 ). Fast segmentation and high-quality three-dimensional volume mesh creation from medical images for diffuse optical tomography . Journal of Biomedical Optics , 18 ( 8 ), 86007 . 10.1117/1.JBO.18.8.086007 23942632 PMC3739873

[IMAG.a.54-b46] Kay , K. N. , Naselaris , T. , Prenger , R. J. , & Gallant , J. L. ( 2008 ). Identifying natural images from human brain activity . Nature , 452 ( 7185 ), 352 – 355 . 10.1038/nature06713 18322462 PMC3556484

[IMAG.a.54-b47] Kell , C. A. , Darquea , M. , Behrens , M. , Cordani , L. , Keller , C. , & Fuchs , S. ( 2017 ). Phonetic detail and lateralization of reading-related inner speech and of auditory and somatosensory feedback processing during overt reading . Human Brain Mapping , 38 ( 1 ), 493 – 508 . 10.1002/hbm.23398 27622923 PMC6866884

[IMAG.a.54-b48] LeBel , A. , Wagner , L. , Jain , S. , Adhikari-Desai , A. , Gupta , B. , Morgenthal , A. , Tang , J. , Xu , L. , & Huth , A. G. ( 2023 ). A natural language fMRI dataset for voxelwise encoding models . Scientific Data , 10 ( 1 ), 555 – 555 . 10.1038/s41597-023-02437-z 37612332 PMC10447563

[IMAG.a.54-b49] Liu , Q. , Farahibozorg , S. , Porcaro , C. , Wenderoth , N. , & Mantini , D. ( 2017 ). Detecting large-scale networks in the human brain using high-density electroencephalography . Human Brain Mapping , 38 ( 9 ), 4631 – 4643 . 10.1002/hbm.23688 28631281 PMC6867042

[IMAG.a.54-b50] Marino , M. , & Mantini , D. ( 2024 ). Human brain imaging with high-density electroencephalography: Techniques and applications . The Journal of Physiology , Advance Publication. 10.1113/jp286639 PMC12810243 39173191

[IMAG.a.54-b51] Markow , Z. E. , Tripathy , K. , Svoboda , A. M. , Schroeder , M. L. , Rafferty , S. M. , Richter , E. J. , Eggebrecht , A. T. , Anastasio , M. A. , Chevillet , M. A. , Mugler , E. M. , Naufel , S. N. , Yin , A. , Trobaugh , J. W. , & Culver , J. P. ( 2023 ). Identifying naturalistic movies from human brain activity with high-density diffuse optical tomography . bioRxiv . 10.1101/2023.11.27.566650

[IMAG.a.54-b53] Markow , Z. E. , Trobaugh , J. W. , Richter , E. J. , Tripathy , K. , Rafferty , S. M. , Svoboda , A. M. , Schroeder , M. L. , Burns-Yocum , T. M. , Bergonzi , K. M. , Chevillet , M. A. , Mugler , E. M. , Eggebrecht , A. T. , & Culver , J. P. ( 2025 ). Ultra high density imaging arrays in diffuse optical tomography for human brain mapping improve image quality and decoding performance . Scientific Reports , 15 ( 1 ), 3175 . 10.1038/s41598-025-85858-7 39863633 PMC11762274

[IMAG.a.54-b87] Matsuda , G. , & Hiraki , K . ( 2006 ). Sustained decrease in oxygenated hemoglobin during video games in the dorsal prefrontal cortex: A NIRS study of children . NeuroImage , 29 ( 3 ), 706 – 711 . 10.1016/j.neuroimage.2005.08.019 16230030

[IMAG.a.54-b54] Maureen , A. O. L. , David , A. B. , Britton , C. , & Arjun , G. Y. ( 1995 ). Simultaneous scattering and absorption images of heterogeneous media using diffusive waves within the Rytov approximation . In Proc.SPIE 2389, Optical Tomography, Photon Migration, and Spectroscopy of Tissue and Model Media: Theory, Human Studies, and Instrumentation, 30 May . SPIE . 10.1117/12.209981

[IMAG.a.54-b55] Mazzonetto , I. , Castellaro , M. , Cooper , R. J. , & Brigadoi , S. ( 2022 ). Smartphone-based photogrammetry provides improved localization and registration of scalp-mounted neuroimaging sensors . Scientific Reports , 12 ( 1 ), 10862 . 10.1038/s41598-022-14458-6 35760834 PMC9237074

[IMAG.a.54-b56] Nishimoto , S. , Vu , A. T. , Naselaris , T. , Benjamini , Y. , Yu , B. , & Gallant , J. L. ( 2011 ). Reconstructing visual experiences from brain activity evoked by natural movies . Current Biology , 21 ( 19 ), 1641 – 1646 . 10.1016/j.cub.2011.08.031 21945275 PMC3326357

[IMAG.a.54-b57] O’Brien , W. J. , Carlton , L. , Muhvich , J. , Kura , S. , Ortega-Martinez , A. , Dubb , J. , Duwadi , S. , Hazen , E. , Yücel , M. A. , von Lühmann , A. , Boas , D. A. , & Zimmermann , B. B. ( 2024 ). ninjaNIRS: An open hardware solution for wearable whole-head high-density functional near-infrared spectroscopy . Biomedical Optics Express , 15 ( 10 ), 5625 – 5644 . 10.1364/BOE.531501 39421779 PMC11482177

[IMAG.a.54-b58] Perdue , K. L. , Jensen , S. K. G. , Kumar , S. , Richards , J. E. , Kakon , S. H. , Haque , R. , Petri , W. A. , Lloyd-Fox , S. , Elwell , C. , & Nelson , C. A. ( 2019 ). Using functional near-infrared spectroscopy to assess social information processing in poor urban Bangladeshi infants and toddlers . Developmental Science , 22 ( 5 ), e12839 . 10.1111/desc.12839 31017372 PMC6737924

[IMAG.a.54-b59] Piper , S. K. , Krueger , A. , Koch , S. P. , Mehnert , J. , Habermehl , C. , Steinbrink , J. , Obrig , H. , & Schmitz , C. H. ( 2014 ). A wearable multi-channel fNIRS system for brain imaging in freely moving subjects . NeuroImage , 85 , 64 – 71 . 10.1016/j.neuroimage.2013.06.062 23810973 PMC3859838

[IMAG.a.54-b60] Poulsen , A. T. , Kamronn , S. , Dmochowski , J. , Parra , L. C. , & Hansen , L. K. ( 2017 ). EEG in the classroom: Synchronised neural recordings during video presentation . Scientific Reports , 7 , 43916 . 10.1038/srep43916 28266588 PMC5339684

[IMAG.a.54-b61] Raschle , N. , Zuk , J. , Ortiz-Mantilla , S. , Sliva , D. D. , Franceschi , A. , Grant , P. E. , Benasich , A. A. , & Gaab , N. ( 2012 ). Pediatric neuroimaging in early childhood and infancy: Challenges and practical guidelines . Annals of the New York Academy of Sciences , 1252 ( 1 ), 43 – 50 . 10.1111/j.1749-6632.2012.06457.x 22524338 PMC3499030

[IMAG.a.54-b62] Roberts , G. , Holmes , N. , Alexander , N. , Boto , E. , Leggett , J. , Hill , R. M. , Shah , V. , Rea , M. , Vaughan , R. , Maguire , E. A. , Kessler , K. , Beebe , S. , Fromhold , M. , Barnes , G. R. , Bowtell , R. , & Brookes , M. J. ( 2019 ). Towards OPM-MEG in a virtual reality environment . NeuroImage , 199 , 408 – 417 . 10.1016/j.neuroimage.2019.06.010 31173906 PMC8276767

[IMAG.a.54-b63] Robinson , A. K. , Venkatesh , P. , Boring , M. J. , Tarr , M. J. , Grover , P. , & Behrmann , M. ( 2017 ). Very high density EEG elucidates spatiotemporal aspects of early visual processing . Scientific Reports , 7 ( 1 ), 16248 . 10.1038/s41598-017-16377-3 29176609 PMC5701165

[IMAG.a.54-b64] Rybář , M. , Poli , R. , & Daly , I. ( 2021 ). Decoding of semantic categories of imagined concepts of animals and tools in fNIRS . Journal of Neural Engineering , 18 ( 4 ), 046035 . 10.1088/1741-2552/abf2e5 33780916

[IMAG.a.54-b65] Schmitz , C. H. , Graber , H. L. , Luo , H. , Arif , I. , Hira , J. , Pei , Y. , Bluestone , A. , Zhong , S. , Andronica , R. , Soller , I. , Ramirez , N. , Barbour , S.-L. S. , & Barbour , R. L. ( 2000 ). Instrumentation and calibration protocol for imaging dynamic features in dense-scattering media by optical tomography . Applied Optics , 39 ( 34 ), 6466 – 6486 . 10.1364/AO.39.006466 18354661

[IMAG.a.54-b66] Schroeder , M. L. , Sherafati , A. , Ulbrich , R. L. , Wheelock , M. D. , Svoboda , A. M. , Klein , E. D. , George , T. G. , Tripathy , K. , Culver , J. P. , & Eggebrecht , A. T. ( 2023 ). Mapping cortical activations underlying covert and overt language production using high-density diffuse optical tomography . NeuroImage , 276 , 120190 . 10.1016/j.neuroimage.2023.120190 37245559 PMC10760405

[IMAG.a.54-b67] Sonkusare , S. , Breakspear , M. , & Guo , C. ( 2019 ). Naturalistic stimuli in neuroscience: Critically acclaimed . Trends in Cognitive Sciences , 23 ( 8 ), 699 – 714 . 10.1016/j.tics.2019.05.004 31257145

[IMAG.a.54-b88] Tachtsidis , I. , & Papaioannou , A. ( 2013 ). Investigation of frontal lobe activation with fNIRS and systemic changes during video gaming . In: S. Van Huffel , G. Naulaers , A. Caicedo , D. F. Bruley , & D. K. Harrison (eds). Oxygen transport to tissue XXXV. Advances in experimental medicine and biology (vol. 789 , pp. 89 – 95 ). Springer. 10.1007/978-1-4614-7411-1_13 PMC4038001 23852481

[IMAG.a.54-b68] Tang , J. , LeBel , A. , Jain , S. , & Huth , A. G. ( 2023 ). Semantic reconstruction of continuous language from non-invasive brain recordings . Nature Neuroscience , 26 ( 5 ), 858 – 866 . 10.1038/s41593-023-01304-9 37127759 PMC11304553

[IMAG.a.54-b69] Tripathy , K. , Fogarty , M. , Svoboda , A. M. , Schroeder , M. L. , Rafferty , S. M. , Richter , E. J. , Tracy , C. , Mansfield , P. K. , Booth , M. , Fishell , A. K. , Sherafati , A. , Markow , Z. E. , Wheelock , M. D. , Arbeláez , A. M. , Schlaggar , B. L. , Smyser , C. D. , Eggebrecht , A. T. , & Culver , J. P. ( 2024 ). Mapping brain function in adults and young children during naturalistic viewing with high-density diffuse optical tomography . Human Brain Mapping , 45 ( 7 ), e26684 . 10.1002/hbm.26684 38703090 PMC11069306

[IMAG.a.54-b70] Tripathy , K. , Markow , Z. E. , Fishell , A. K. , Sherafati , A. , Burns-Yocum , T. M. , Schroeder , M. L. , Svoboda , A. M. , Eggebrecht , A. T. , Anastasio , M. A. , Schlaggar , B. L. , & Culver , J. P. ( 2021 ). Decoding visual information from high-density diffuse optical tomography neuroimaging data . NeuroImage , 226 , 117516 . 10.1016/j.neuroimage.2020.117516 33137479 PMC8006181

[IMAG.a.54-b71] Tripathy , K. , Markow , Z. E. , Fogarty , M. , Schroeder , M. L. , Svoboda , A. M. , Eggebrecht , A. , Schlaggar , B. L. , Trobaugh , J. , & Culver , J. ( 2025 ). Multisensory naturalistic decoding with high-density diffuse optical tomography . Neurophotonics , 12 ( 1 ), 015002 . 10.1117/1.NPh.12.1.015002 39850351 PMC11755382

[IMAG.a.54-b72] Tsao , D. Y. , & Livingstone , M. S. ( 2008 ). Mechanisms of face perception . Annual Review of Neuroscience , 31 ( 1 ), 411 – 437 . 10.1146/annurev.neuro.30.051606.094238 PMC2629401 18558862

[IMAG.a.54-b73] Uchitel , J. , Blanco , B. , Vidal-Rosas , E. , Collins-Jones , L. , & Cooper , R. J. ( 2022 ). Reliability and similarity of resting state functional connectivity networks imaged using wearable, high-density diffuse optical tomography in the home setting . NeuroImage , 263 , 119663 . 10.1016/j.neuroimage.2022.119663 36202159

[IMAG.a.54-b74] Vanderwal , T. , Eilbott , J. , & Castellanos , F. X. ( 2019 ). Movies in the magnet: Naturalistic paradigms in developmental functional neuroimaging . Developmental Cognitive Neuroscience , 36 , 100600 . 10.1016/j.dcn.2018.10.004 30551970 PMC6969259

[IMAG.a.54-b75] Vanzella , P. , Balardin , J. B. , Furucho , R. A. , Zimeo Morais , G. A. , Braun Janzen , T. , Sammler , D. , & Sato , J. R. ( 2019 ). fNIRS responses in professional violinists while playing duets: Evidence for distinct leader and follower roles at the brain level . Frontiers in Psychology , 10 , 164 . 10.3389/fpsyg.2019.00164 30804846 PMC6370678

[IMAG.a.54-b76] Vidal-Rosas , E. , Zhao , H. , Nixon-Hill , R. , Smith , G. , Dunne , L. , Powell , S. , Cooper , R. , & Everdell , N. ( 2021 ). Evaluating a new generation of wearable high-density diffuse optical tomography technology via retinotopic mapping of the adult visual cortex . Neurophotonics , 8 ( 2 ), 025002 . 10.1117/1.NPh.8.2.025002 33842667 PMC8033536

[IMAG.a.54-b77] Vidal-Rosas , E. E. , Hill , R. , Smith , G. , Dunne , L. , Zhao , H. , Powell , S. , Everdell , N. L. , & Cooper , R. J. ( 2020 ). Wearable high-density diffuse optical tomography (HD-DOT) for unrestricted 3D functional neuroimaging . In Biophotonics Congress: Biomedical Optics 2020 (Translational, Microscopy, OCT, OTS, BRAIN), Washington, DC (OSA Technical Digest). Optica Publishing Group . 10.1364/ots.2020.stu1d.3

[IMAG.a.54-b78] Vidal-Rosas , E. E. , von Lühmann , A. , Pinti , P. , & Cooper , R. J. ( 2023 ). Wearable, high-density fNIRS and diffuse optical tomography technologies: A perspective . Neurophotonics , 10 ( 2 ), 023513 . 10.1117/1.NPh.10.2.023513 37207252 PMC10190166

[IMAG.a.54-b79] Wheelock , M. D. , Culver , J. P. , & Eggebrecht , A. T. ( 2019 ). High-density diffuse optical tomography for imaging human brain function . Review of Scientific Instruments , 90 ( 5 ), 051101 . 10.1063/1.5086809 31153254 PMC6533110

[IMAG.a.54-b80] White , B. R. , & Culver , J. P. ( 2010 ). Quantitative evaluation of high-density diffuse optical tomography: In vivo resolution and mapping performance . Journal of Biomedical Optics , 15 , 026006 . 10.1117/1.3368999 20459251 PMC2874047

[IMAG.a.54-b81] Woolnough , O. , Donos , C. , Curtis , A. , Rollo , P. S. , Roccaforte , Z. J. , Dehaene , S. , Fischer-Baum , S. , & Tandon , N. ( 2021 ). A spatiotemporal map of reading aloud . Cold Spring Harbor Laboratory . 10.1523/JNEUROSCI.2324-21.2022 PMC9270918 35641189

[IMAG.a.54-b82] Yoo , S. H. , Santosa , H. , Kim , C. S. , & Hong , K. S. ( 2021 ). Decoding multiple sound-categories in the auditory cortex by neural networks: An fNIRS study . Frontiers in Human Neuroscience , 15 , 636191 . 10.3389/fnhum.2021.636191 33994978 PMC8113416

[IMAG.a.54-b83] Yucel , M. A. , Luhmann , A. V. , Scholkmann , F. , Gervain , J. , Dan , I. , Ayaz , H. , Boas , D. , Cooper , R. J. , Culver , J. , Elwell , C. E. , Eggebrecht , A. , Franceschini , M. A. , Grova , C. , Homae , F. , Lesage , F. , Obrig , H. , Tachtsidis , I. , Tak , S. , Tong , Y. ,… Wolf , M . ( 2021 ). Best practices for fNIRS publications . Neurophotonics , 8 ( 1 ), 012101 . 10.1117/1.NPh.8.1.012101 33442557 PMC7793571

[IMAG.a.54-b84] Zeff , B. W. , White , B. R. , Dehghani , H. , Schlaggar , B. L. , & Culver , J. P. ( 2007 ). Retinotopic mapping of adult human visual cortex with high-density diffuse optical tomography . Proceedings of the National Academy of Sciences of the United States of America , 104 ( 29 ), 12169 – 12174 . 10.1073/pnas.0611266104 17616584 PMC1924577

[IMAG.a.54-b85] Zinszer , B. D. , Bayet , L. , Emberson , L. L. , Raizada , R. D. S. , & Aslin , R. N. ( 2018 ). Decoding semantic representations from functional near-infrared spectroscopy signals . Neurophotonics , 5 ( 1 ), 011003 . 10.1117/1.NPh.5.1.011003 28840167 PMC5568915

