# Supplementary material for: Functional brain mapping using whole-head very high-density diffuse optical tomography
Source: Imaging Neurosci (Camb). 2025 Jun 20;3:IMAG.a.54. doi: 10.1162/IMAG.a.54 (PMC12320020; doi:10.1162/IMAG.a.54)
Supplement: Supplementary Material [file imag.a.54_supp.pdf]

Supplemental Methods:

S1 DOT Image Reconstruction (Inverse Problem):

To obtain the changes in absorption within the tissue, the sensitivity matrix, $\boldsymbol{A}$, must be inverted. Tikhonov regularization with an additional spatially variant regularization term was used following prior literature (Eggebrecht et al., 2014; Markow et al., 2023; Tripathy et al., 2024; Zeff et al., 2007). In this case, image reconstruction is equivalent to minimizing the objective function in Eq. 1.

$$\widehat{\boldsymbol{x}} = \text{argmin}_{x}\{||\boldsymbol{y} - \boldsymbol{A}\boldsymbol{x}||_2^2 + \lambda_1||\boldsymbol{L}\boldsymbol{x}||_2^2\} \qquad \text{Eq. 1}$$

The tradeoff between noise and spatial resolution is controlled by the Tikhonov regularization parameter (λ_1). The $\boldsymbol{L}$ term is a diagonal matrix for providing spatially variant regularization (Eq. 2). The spatially variant regularization parameter (λ_2) reduces spatial localization errors induced by Tikhonov regularization biasing images towards the surface of the head.

$$\text{diag}(\boldsymbol{L}) = \sqrt[2]{\text{diag}(\boldsymbol{A}^T\boldsymbol{A})} + \lambda_2\text{max}(\text{diag}(\boldsymbol{A}^T\boldsymbol{A})) \qquad \text{Eq. 2}$$

A regularized Moore-Penrose pseudoinverse, $\boldsymbol{A}^{\#}_{\lambda_1\lambda_2}$ (Eq. 4), is used to compute the reconstructed image (Eq. 3). Here, $\tilde{s}^2_{\text{max}}$ is the maximum singular value of $\widetilde{\boldsymbol{A}}$ (Eq. 5) which allows interpretation of λ_1 as a fraction of the maximum singular value.

$$\widehat{\boldsymbol{x}} = \boldsymbol{A}^{\#}_{\lambda_1\lambda_2}\boldsymbol{y} \qquad \text{Eq. 3}$$

$$\boldsymbol{A}^{\#}_{\lambda_1\lambda_2} = \boldsymbol{L}^{-1}(\widetilde{\boldsymbol{A}}^T\widetilde{\boldsymbol{A}} + \lambda_1^2\tilde{s}^2_{\text{max}}\boldsymbol{I})^{-1}\widetilde{\boldsymbol{A}}^T \qquad \text{Eq. 4}$$

$$\widetilde{\boldsymbol{A}} = \boldsymbol{A}\boldsymbol{L}^{-1} \qquad \text{Eq. 5}$$

S2: VHD-DOT Instrumentation and Design

A dedicated computer with custom software for the illumination patterns controls the light sources. Following previous system designs, four sets of 64 sources are connected to a 20 MHz digital input/output card (PCIe-6537B Part #782608-01, National Instruments) and custom "brain boxes" for multiplexing and demultiplexing signals (Markow et al., 2023). For the two-pass encoding pattern, sources are illuminated at a 50% duty cycle for the first pass of the encoding scheme and 1% duty cycle for the second pass. This encoding scheme utilizes frequency encoding as light sources are modulated at 13,228 Hz and 19,841 Hz for simultaneous collection at the 685 and 830 nm wavelengths, respectively. Spatial and temporal encoding involves illumination of only one source position at a time within a designated spatial region to ensure that detectors are not illuminated by two high-magnitude source positions simultaneously, while spatial encoding allows simultaneous illumination in regions with minimal optical crosstalk.

Signals from each of the 252 avalanche photodiode detectors are digitized using analog-to-digital converters (ADC) at 96 kHz (Focusrite RedNet A16R MkII). Two computers, one for detectors 1-126 and another for detectors 127-252, with custom software display real-time light and noise

levels for the digitized signals, are used for recording during data collection. To avoid detector saturation and allow the analog-to-digital converters to settle between measurements, a 104μs off period is included in the temporal encoding pattern. A diagram of the system configuration is included in **Supplemental Fig. 1**.

To manage the 507 cables connected to the imaging cap, both source and detector fibers were bundled separately in groups of 130 fibers to the left and right side panels, 120 to the visual panel, and 127 to the motor pad. Fibers were separated into smaller bundles of 5-6 fibers, corresponding to each cap column. This allowed for organization of the fibers around the concentric wooden halos and the motor pad.

S3 MRI Preprocessing:

The following fMIR preprocessing methods are derived from the fMRIPrep boilerplate text as recommended for citing fMRIPrep.

S3.1 Preprocessing of B0 inhomogeneity mappings: Each subject had at least one field map per imaging session. A B0-nonuniformity map (or fieldmap) was estimated based on two (or more) echo-planar imaging (EPI) references with topup (Andersson et al., 2003); FSL 6.0.5.1:57b01774).

S3.2 Anatomical data preprocessing: A total of 1 T1-weighted (T1w) images were found per subject within the input BIDS dataset. The T1-weighted (T1w) image was corrected for intensity non-uniformity (INU) with N4BiasFieldCorrection (Tustison et al., 2010), distributed with ANTs 2.3.3 ((Avants et al., 2008), RRID:SCR_004757), and used as T1w-reference throughout the workflow. The T1w-reference was then skull-stripped with a Nipype implementation of the antsBrainExtraction.sh workflow (from ANTs), using OASIS30ANTs as target template. Brain tissue segmentation of cerebrospinal fluid (CSF), white-matter (WM) and gray-matter (GM) was performed on the brain-extracted T1w using fast (FSL 6.0.5.1:57b01774, RRID:SCR_002823, (Zhang et al., 2001). Brain surfaces were reconstructed using recon-all (FreeSurfer 7.2.0, RRID:SCR_001847, (Dale et al., 1999), and the brain mask estimated previously was refined with a custom variation of the method to reconcile ANTs-derived and FreeSurfer-derived segmentations of the cortical gray-matter of Mindboggle (RRID:SCR_002438, (Klein et al., 2017). Volume-based spatial normalization to one standard space (MNI152NLin2009cAsym) was performed through nonlinear registration with antsRegistration (ANTs 2.3.3), using brain-extracted versions of both T1w reference and the T1w template. The following template was selected for spatial normalization: ICBM 152 Nonlinear Asymmetrical template version 2009c [(Fonov et al., 2009), RRID:SCR_008796; TemplateFlow ID: MNI152NLin2009cAsym].

S3.3 Functional data preprocessing: For each of the BOLD runs found per subject (across all tasks and sessions), the following preprocessing was performed. First, a reference volume and its skull-stripped version were generated by aligning and averaging 1 single-band references (SBRefs). Head-motion parameters with respect to the BOLD reference (transformation matrices, and six corresponding rotation and translation parameters) are estimated before any spatiotemporal filtering using mcflirt (FSL 6.0.5.1:57b01774, (Jenkinson et al., 2002)). The estimated fieldmap was then aligned with rigid-registration to the target EPI (echo-planar imaging) reference run. The field coefficients were mapped on to the reference EPI using the transform. BOLD runs were slice-time corrected to 0.566s (0.5 of slice acquisition range 0s-1.13s) using 3dTshift from AFNI (Cox & Hyde, 1997), RRID:SCR_005927). The BOLD reference was then co-registered to the T1w reference using bbregister (FreeSurfer) which implements boundary-based

registration (Greve & Fischl, 2009). Co-registration was configured with six degrees of freedom. First, a reference volume and its skull-stripped version were generated using a custom methodology of fMRIPrep. Several confounding time-series were calculated based on the preprocessed BOLD: framewise displacement (FD), DVARS and three region-wise global signals. FD was computed using two formulations following Power (absolute sum of relative motions, (Power et al., 2014)) and Jenkinson (relative root mean square displacement between affines, (Jenkinson et al., 2002)). FD and DVARS are calculated for each functional run, both using their implementations in Nipype (following the definitions by (Power et al., 2014). The three global signals are extracted within the CSF, the WM, and the whole-brain masks. Additionally, a set of physiological regressors were extracted to allow for component-based noise correction (CompCor, (Behzadi et al., 2007)). Principal components are estimated after high-pass filtering the preprocessed BOLD time-series (using a discrete cosine filter with 128s cut-off) for the two CompCor variants: temporal (tCompCor) and anatomical (aCompCor). tCompCor components are then calculated from the top 2% variable voxels within the brain mask. For aCompCor, three probabilistic masks (CSF, WM and combined CSF+WM) are generated in anatomical space. The implementation differs from that of Behzadi et al. in that instead of eroding the masks by 2 pixels on BOLD space, a mask of pixels that likely contain a volume fraction of GM is subtracted from the aCompCor masks. This mask is obtained by dilating a GM mask extracted from the FreeSurfer's aseg segmentation, and it ensures components are not extracted from voxels containing a minimal fraction of GM. Finally, these masks are resampled into BOLD space and binarized by thresholding at 0.99 (as in the original implementation). Components are also calculated separately within the WM and CSF masks. For each CompCor decomposition, the k components with the largest singular values are retained, such that the retained components' time series are sufficient to explain 50 percent of variance across the nuisance mask (CSF, WM, combined, or temporal). The remaining components are dropped from consideration. The head-motion estimates calculated in the correction step were also placed within the corresponding confounds file. The confound time series derived from head motion estimates and global signals were expanded with the inclusion of temporal derivatives and quadratic terms for each (Satterthwaite et al., 2013). Frames that exceeded a threshold of 0.5 mm FD or 1.5 standardized DVARS were annotated as motion outliers. Additional nuisance timeseries are calculated by means of principal components analysis of the signal found within a thin band (crown) of voxels around the edge of the brain, as proposed by (Patriat et al., 2017). The BOLD time-series were resampled into standard space, generating a preprocessed BOLD run in MNI152NLin2009cAsym space. First, a reference volume and its skull-stripped version were generated using a custom methodology of fMRIPrep. All resamplings can be performed with a single interpolation step by composing all the pertinent transformations (i.e. head-motion transform matrices, susceptibility distortion correction when available, and co-registrations to anatomical and output spaces). Gridded (volumetric) resamplings were performed using antsApplyTransforms (ANTs), configured with Lanczos interpolation to minimize the smoothing effects of other kernels (Lanczos, 1964). Non-gridded (surface) resamplings were performed using mri_vol2surf (FreeSurfer).

Supplemental Figures and Tables

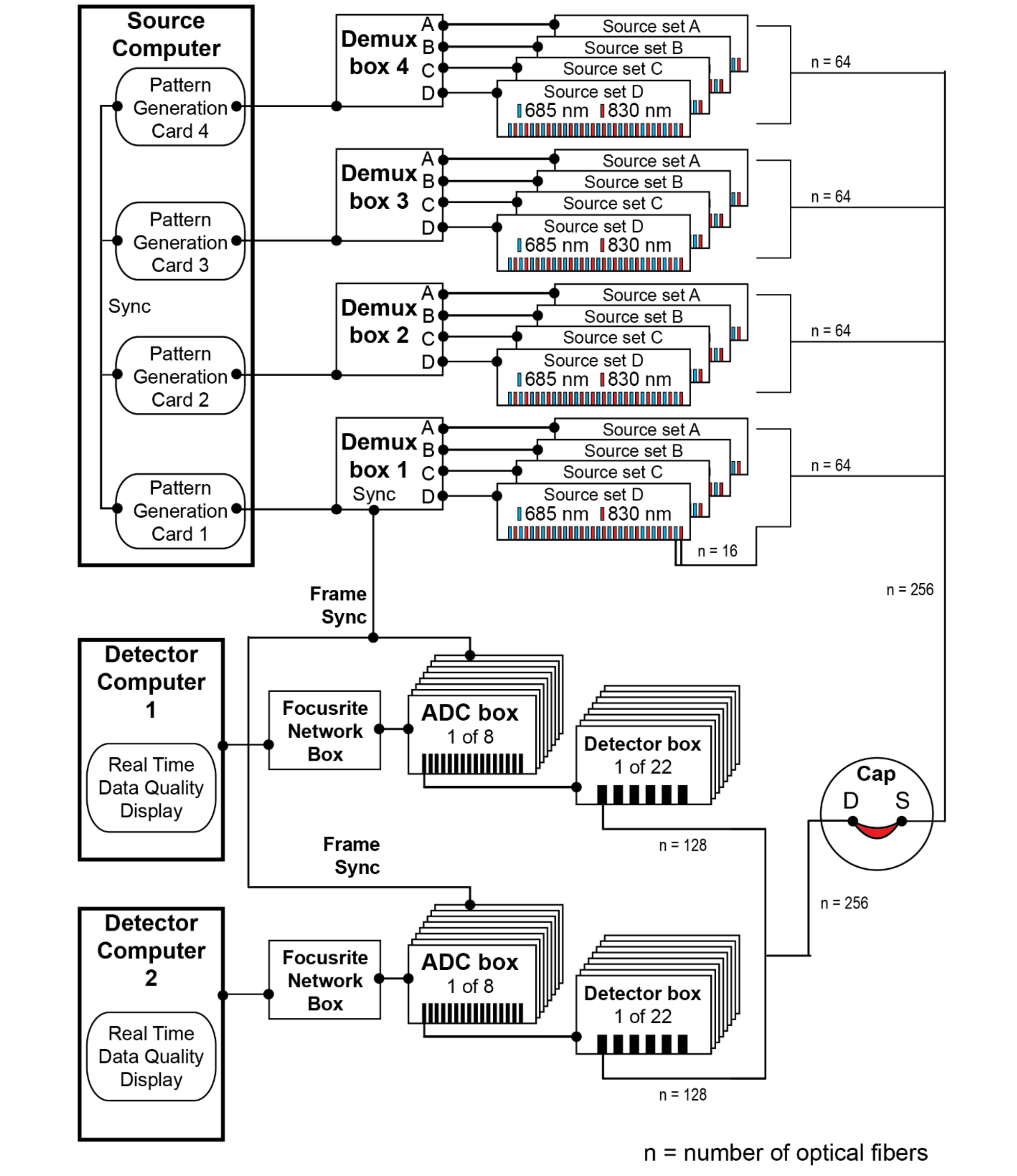

Supplemental Figure 1: Schematic of VHD-DOT imaging system. Sources are illuminated following the spatial, temporal, and frequency encoding patterns from the source computer. Light is transported to the participant using 252 fiber optic cables embedded in the imaging cap. Light is similarly transported using 255 fiber optic cables to the avalanche photodiode detectors and analog-to-digital converters, which are controlled by the two detector computers. The number of optical fibers is denoted as ‘n’ in the figure to illustrate the number of fibers connected to each source and detector box.

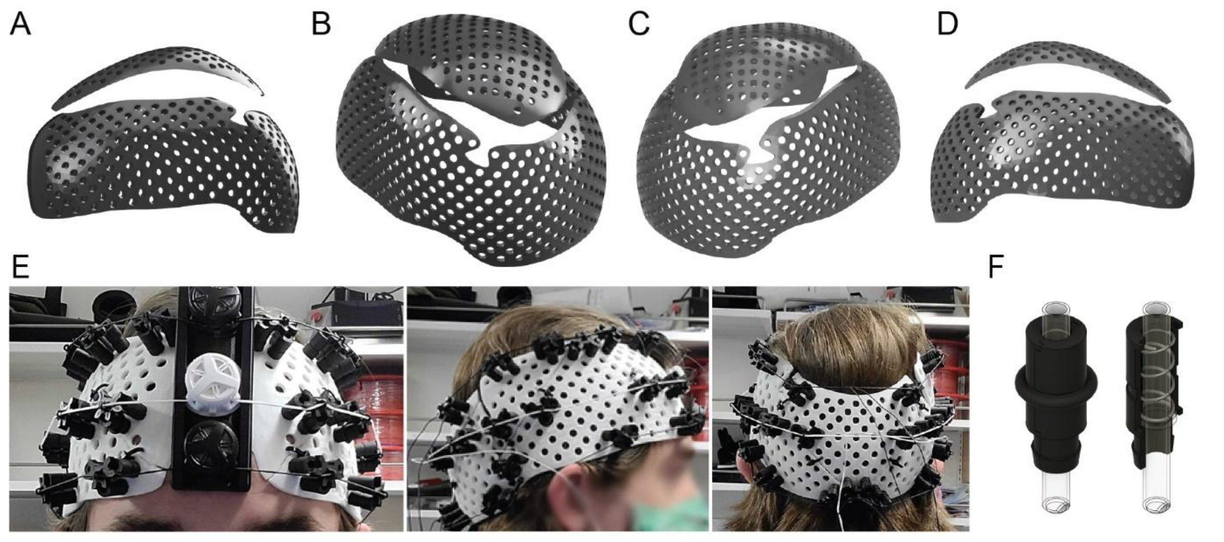

Supplemental Figure 2: The VHD-DOT cap was designed in Autodesk Fusion 360 using a mesh generated from the structural MRI scan on a representative participant (A-D). The cap model was populated with 6.25 mm diameter holes for the fiber housing. A ratchet mechanism (E) was introduced to evenly distribute pressure across the participant's head. Three ratchets were used to tighten the cap, similar to a bike helmet. Spring-loaded fiber tip housings (F) were designed and 3D printed to allow the cap to conform to various head shapes and sizes while additionally reducing any discomfort from individual optodes. These cap improvements maximize the optode-to-scalp coupling necessary for high-quality DOT data.

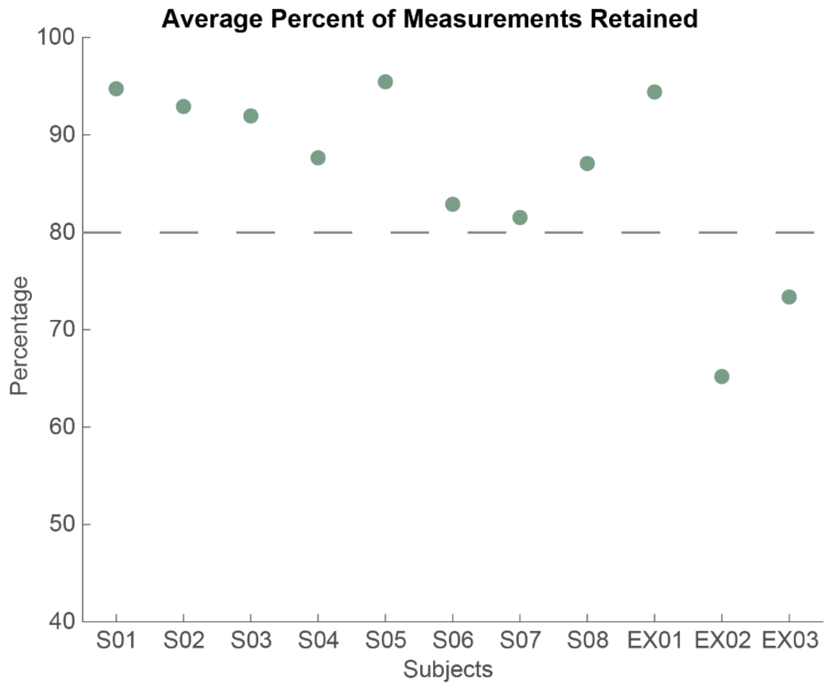

Supplemental Figure 3: Participant retention was based on the average percentage of retained measurements across all runs within each participant. Participants were excluded if their measurement percentage was less than 80%, indicating a poor cap fit. Three participants were excluded from the study with two participants excluded based on the retained measurements. Participant EX01 was excluded due to an insufficient amount of fMRI data collected during their scan session.

	**VHD-DOT Runs**					**MRI Runs**				
**Subject**	**Hearing Words**	**Generate Verbs**	**Retinotopy**	**Finger Tapping**	**Movie Viewing**	**Hearing Words**	**Generate Verbs**	**Retinotopy**	**Finger Tapping**	**Movie Viewing**
S01	3	2	2	2	2	2	2	2	2	2
S02	3	2	1	2	2	2	2	2	2	2
S03	3	1	2	2	2	2	2	2	2	2
S04	4	2	2	2	2	2	2	2	2	2
S05	3	2	2	2	2	2	2	2	2	2
S06	3	2	2	2	2	2	2	2	2	2
S07	3	2	2	1	2	2	2	2	2	2
S08	3	2	2	2	2	2	2	2	2	2
Total:	25	15	15	15	16	16	16	16	16	16

Supplemental Table 1: This table contains the total number of runs collected in each of the 8 participants and included in the data analysis. The VHD-DOT data were collected across two sessions and the MRI data were collected in a single session.

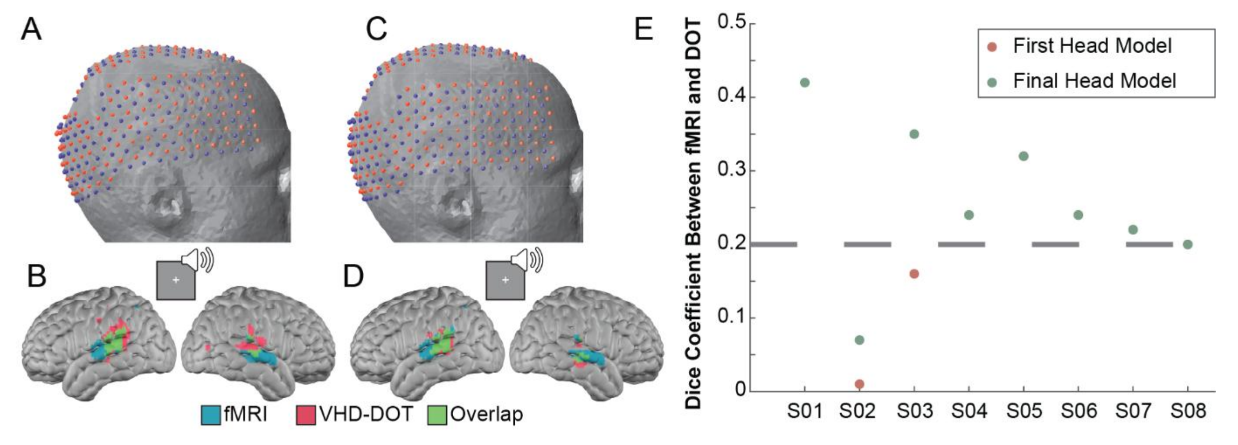

Supplemental Figure 4: Subject-specific light modeling requires the precise placement of the VHD-DOT optode grid onto the head mesh of each individual participant. Here, the first iteration of the cap placement for a participant (A) is shown with the corresponding word hearing task activation map from this light model iteration (B). The overlap between the subject's word hearing MRI data (ground truth) and the VHD-DOT data is quantified using the Dice coefficient. Since this Dice coefficient is less than 0.2, additional light model iterations were generated until the Dice coefficient exceeded 0.2 (C-D). This was completed for each participant (E) in order to generate the subject-specific light models.

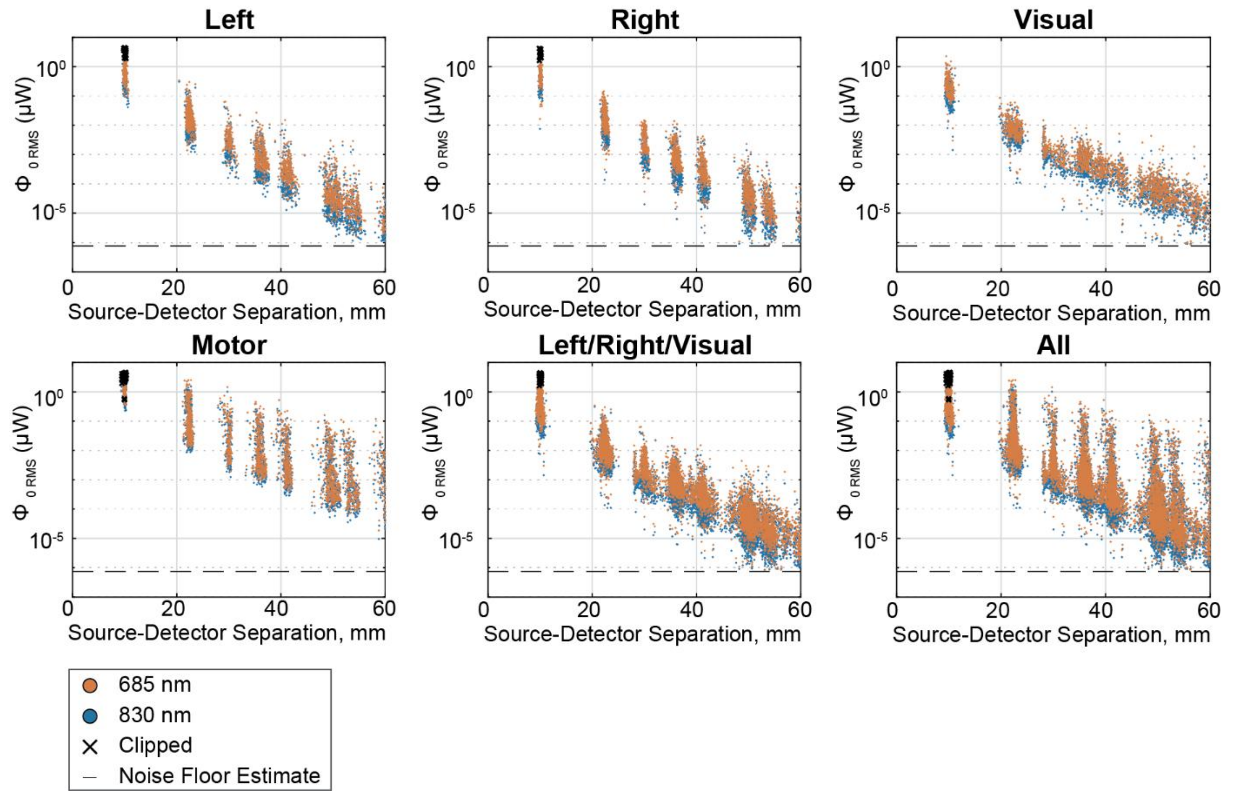

Supplemental Figure 5: The light falloff curves represent the light intensity as a function of source-detector separation with each measurement representing a point on the plots for both wavelengths. The sections of the cap were divided between the left, right, visual (or back), and the motor (or top)

panels. The left, right, and visual panels were combined to represent the majority of the cap without the motor panel. The All plot combines the plots from each subsection of the cap.

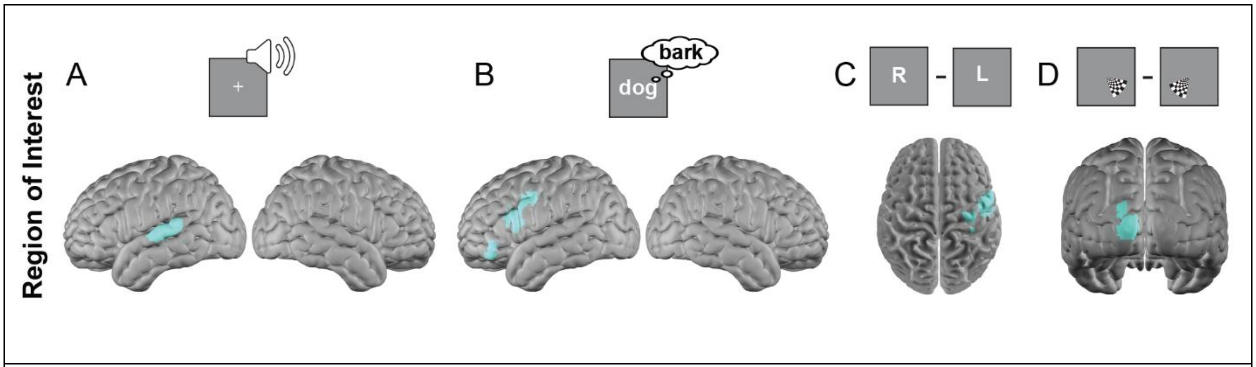

Supplemental Figure 6: Regions of interest for the oxy-, deoxy-, and total hemoglobin and BOLD signal plots in main text Figure 3. These regions were generated for each task based on thresholding the group average fixed effect t-statistic maps at 50% of the maximum value.

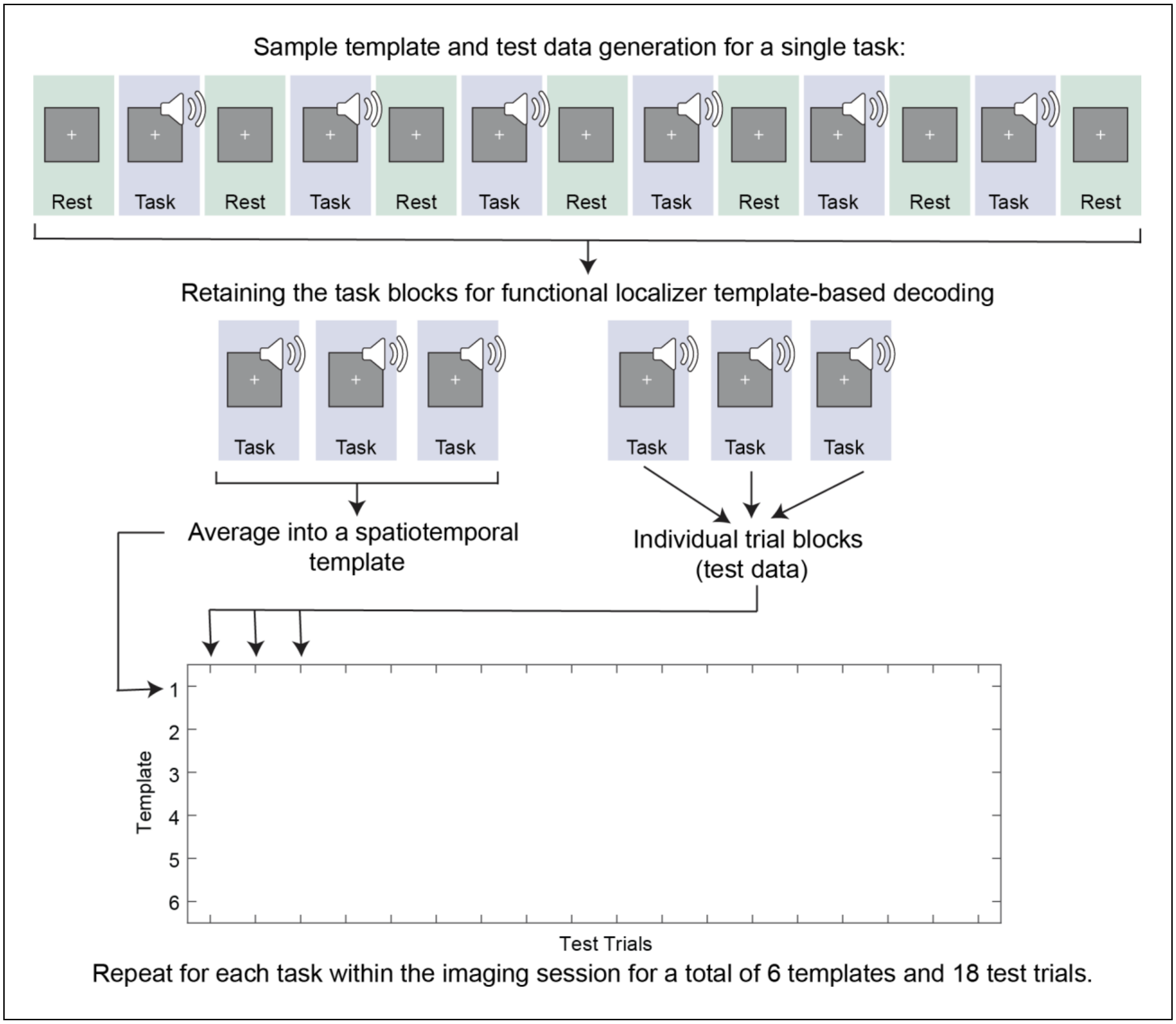

Supplemental Figure 7: Template-based decoding design for functional localizers using the auditory task as a representative task. This task consisted of 6 blocks of hearing words and seven blocks of rest. The 6 task blocks were divided into three blocks for training and three blocks for testing. The three training blocks were averaged together to generate a spatiotemporal template, while the testing blocks were left as individual trial blocks. This was repeated for each task in the imaging session for a total of 6 templates and 18 testing blocks. The spatiotemporal correlation between the template and the testing blocks was computed for each pair of template and training block and arranged in a grid as depicted in **Fig. 4A**.

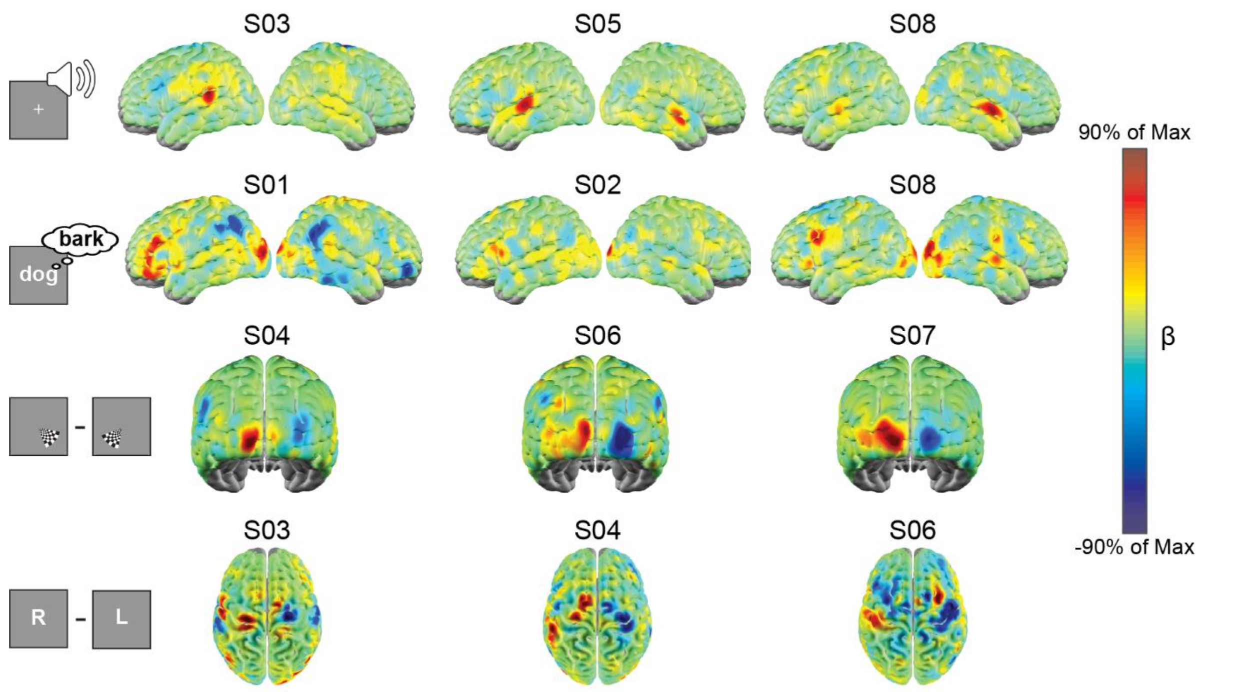

Supplemental Figure 8: Individual oxy-hemoglobin task activation maps for three representative participants for each task. The participant is indicated by the S0X above each map, with each participant represented at least once. These maps indicate that we can achieve reliable task activation maps from single runs.

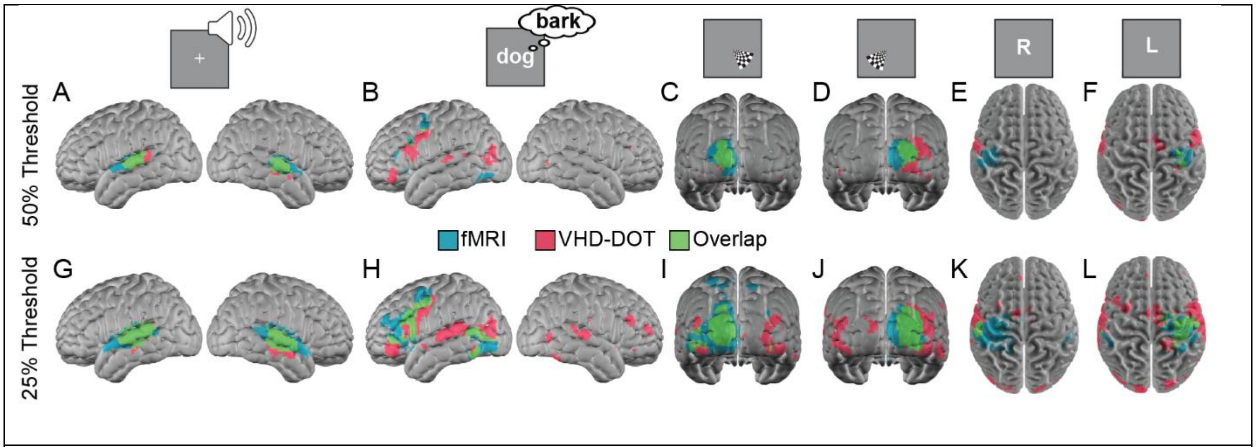

Supplemental Figure 9: Group-average fMRI and VHD-DOT functional localizer maps were binarized using a threshold of 50% (top) or 25% (bottom) of the maximum t-statistic value. These binarized maps were then overlaid to generate overlap maps for the word hearing (A,G), verb generation (B,H), visual right (C,I), visual left (D,J), motor right (E,K), and motor left (F,L) tasks. These maps demonstrate the spatial agreement between the VHD-DOT and fMRI data and highlight the similarities between the two modalities.

Task	25% Threshold Dice Coefficient	50% Threshold Dice Coefficient
**Word Hearing (Auditory)**	0.63	0.54
**Verb Generation (Language)**	0.30	0.06
**Visual Right**	0.22	0.40
**Visual Left**	0.16	0.23
**Motor Right**	0.08	0.00
**Motor Left**	0.19	0.16

Supplemental Table 2: The Dice coefficients between the group-average fMRI and VHD-DOT binarized maps from Supplemental Figure 8 were computed for the 25% and 50% of maximum t-statistic maps. These values quantitatively compare the VHD-DOT and fMRI maps for each of the functional localizer tasks.

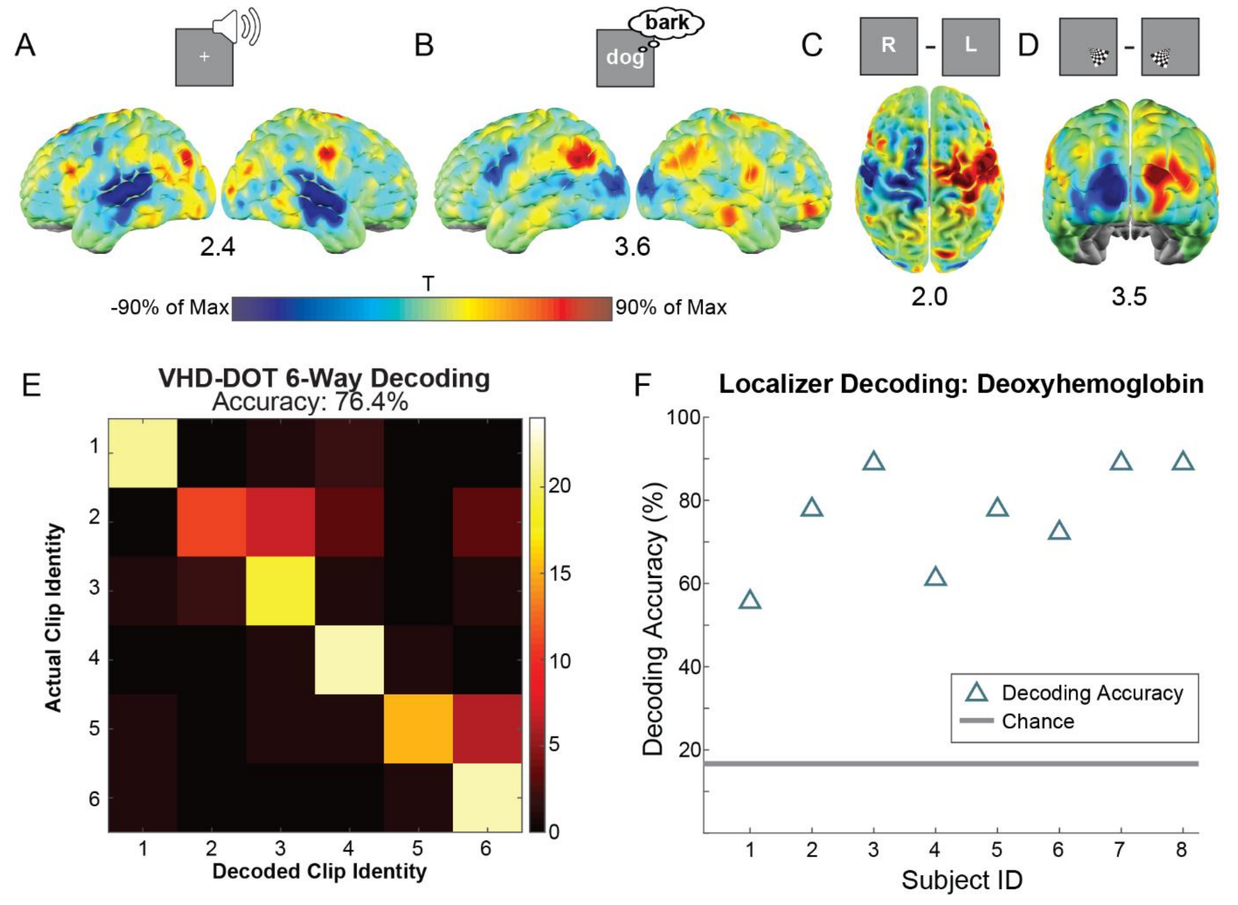

Supplemental Figure 10: Fixed effect t-statistic group activation maps for word hearing (A), verb generation (B), finger tapping (C), and visual (D) from deoxyhemoglobin. These maps appear similar to those presented in Figure 3 for oxy-hemoglobin but are inverted with deoxy-hemoglobin as the inverse of oxy-hemoglobin. The six-way decoding task using deoxyhemoglobin resulted in similar decoding performance when aggregated across subjects (E), and within individual subjects (F). This indicates that VHD-DOT can be used to measure not only oxy-hemoglobin but also deoxy-hemoglobin with considerable accuracy.

Supplemental References:

Andersson, J. L. R., Skare, S., & Ashburner, J. (2003). How to correct susceptibility distortions in spin-echo echo-planar images: application to diffusion tensor imaging. *NeuroImage*, *20*(2), 870-888. https://doi.org/10.1016/S1053-8119(03)00336-7

Avants, B. B., Epstein, C. L., Grossman, M., & Gee, J. C. (2008). Symmetric diffeomorphic image registration with cross-correlation: Evaluating automated labeling of elderly and neurodegenerative brain. *Medical Image Analysis*, *12*(1), 26-41. https://doi.org/https://doi.org/10.1016/j.media.2007.06.004

Behzadi, Y., Restom, K., Liau, J., & Liu, T. T. (2007). A component based noise correction method (CompCor) for BOLD and perfusion based fMRI. *NeuroImage*, *37*(1), 90-101. https://doi.org/10.1016/j.neuroimage.2007.04.042

Cox, R. W., & Hyde, J. S. (1997). Software tools for analysis and visualization of fMRI data. *NMR in Biomedicine*, *10*(4-5), 171-178. https://doi.org/10.1002/(SICI)1099-1492(199706/08)10:4/5<171::AID-NBM453>3.0.CO;2-L

Dale, A. M., Fischl, B., & Sereno, M. I. (1999). Cortical Surface-Based Analysis: I. Segmentation and Surface Reconstruction. *NeuroImage*, *9*(2), 179-194. https://doi.org/https://doi.org/10.1006/nimg.1998.0395

Eggebrecht, A. T., Ferradal, S. L., Robichaux-Viehoever, A., Hassanpour, M. S., Dehghani, H., Snyder, A. Z., Hershey, T., & Culver, J. P. (2014). Mapping distributed brain function and networks with diffuse optical tomography. *Nature Photonics*, *8*(6), 448-454. https://doi.org/10.1038/nphoton.2014.107

Fonov, V. S., Evans, A. C., McKinstry, R. C., Almli, C. R., & Collins, D. L. (2009). Unbiased nonlinear average age-appropriate brain templates from birth to adulthood. *NeuroImage*, *47, Supplement 1*, S102-S102. https://doi.org/10.1016/S1053-8119(09)70884-5

Greve, D. N., & Fischl, B. (2009). Accurate and robust brain image alignment using boundary-based registration. *NeuroImage*, *48*(1), 63-72. https://doi.org/10.1016/j.neuroimage.2009.06.060

Jenkinson, M., Bannister, P., Brady, M., & Smith, S. (2002). Improved Optimization for the Robust and Accurate Linear Registration and Motion Correction of Brain Images. *NeuroImage*, *17*(2), 825-841. https://doi.org/10.1006/nimg.2002.1132

Klein, A., Ghosh, S. S., Bao, F. S., Giard, J., Häme, Y., Stavsky, E., Lee, N., Rossa, B., Reuter, M., Neto, E. C., & Keshavan, A. (2017). Mindboggling morphometry of human brains. *PLOS Computational Biology*, *13*(2), e1005350-e1005350. https://doi.org/10.1371/journal.pcbi.1005350

Lanczos, C. (1964). Evaluation of Noisy Data. *Journal of the Society for Industrial and Applied Mathematics Series B Numerical Analysis*, *1*(1), 76-85. https://doi.org/10.1137/0701007

Markow, Z. E., Trobaugh, J. W., Richter, E. J., Tripathy, K., Rafferty, S. M., Svoboda, A. M., Schroeder, M. L., Burns-Yocum, T. M., Bergonzi, K. M., Chevillet, M. A., Mugler, E. M., Eggebrecht, A. T., & Culver, J. P. (2023). Ultra-high density imaging arrays for diffuse optical tomography of human brain improve resolution, signal-to-noise, and information decoding. https://doi.org/10.1101/2023.07.21.549920

Patriat, R., Reynolds, R. C., & Birn, R. M. (2017). An improved model of motion-related signal changes in fMRI. *NeuroImage*, *144, Part A*, 74-82. https://doi.org/10.1016/j.neuroimage.2016.08.051

Power, J. D., Mitra, A., Laumann, T. O., Snyder, A. Z., Schlaggar, B. L., & Petersen, S. E. (2014). Methods to detect, characterize, and remove motion artifact in resting state fMRI. *NeuroImage*, *84*(Supplement C), 320-341. https://doi.org/10.1016/j.neuroimage.2013.08.048

Satterthwaite, T. D., Elliott, M. A., Gerraty, R. T., Ruparel, K., Loughead, J., Calkins, M. E., Eickhoff, S. B., Hakonarson, H., Gur, R. C., Gur, R. E., & Wolf, D. H. (2013). An

improved framework for confound regression and filtering for control of motion artifact in the preprocessing of resting-state functional connectivity data. *NeuroImage*, *64*(1), 240-256. https://doi.org/10.1016/j.neuroimage.2012.08.052

Tripathy, K., Fogarty, M., Svoboda, A. M., Schroeder, M. L., Rafferty, S. M., Richter, E. J., Tracy, C., Mansfield, P. K., Booth, M., Fishell, A. K., Sherafati, A., Markow, Z. E., Wheelock, M. D., Arbeláez, A. M., Schlaggar, B. L., Smyser, C. D., Eggebrecht, A. T., & Culver, J. P. (2024). Mapping brain function in adults and young children during naturalistic viewing with high-density diffuse optical tomography. *Human Brain Mapping*, *45*(7), e26684. https://doi.org/https://doi.org/10.1002/hbm.26684

Tustison, N. J., Avants, B. B., Cook, P. A., Zheng, Y., Egan, A., Yushkevich, P. A., & Gee, J. C. (2010). N4ITK: Improved N3 Bias Correction. *IEEE Transactions on Medical Imaging*, *29*(6), 1310-1320. https://doi.org/10.1109/TMI.2010.2046908

Zeff, B. W., White, B. R., Dehghani, H., Schlaggar, B. L., & Culver, J. P. (2007). Retinotopic mapping of adult human visual cortex with high-density diffuse optical tomography. *Proceedings of the National Academy of Sciences*, *104*(29), 12169-12174. www.pnas.orgcgidoi10.1073pnas.0611266104

Zhang, Y., Brady, M., & Smith, S. (2001). Segmentation of brain MR images through a hidden Markov random field model and the expectation-maximization algorithm. *IEEE Transactions on Medical Imaging*, *20*(1), 45-57. https://doi.org/10.1109/42.906424
